# The Effect of Sound Frequency and Intensity on Yeast Growth, Fermentation Performance and Volatile Composition of Beer

**DOI:** 10.3390/molecules26237239

**Published:** 2021-11-29

**Authors:** Parise Adadi, Alastair Harris, Phil Bremer, Patrick Silcock, Austen R. D. Ganley, Andrew G. Jeffs, Graham T. Eyres

**Affiliations:** 1 Department of Food Science, University of Otago, P.O. Box 56, Dunedin 9054, New Zealand; parise.adadi@postgrad.otago.ac.nz (P.A.); phil.bremer@otago.ac.nz (P.B.); pat.silcock@otago.ac.nz (P.S.); 2 School of Biological Sciences, University of Auckland, Auckland 1142, New Zealand; ahar394@aucklanduni.ac.nz (A.H.); a.ganley@auckland.ac.nz (A.R.D.G.); a.jeffs@auckland.ac.nz (A.G.J.); 3 Institute of Marine Science, University of Auckland, Private Bag, Auckland 92019, New Zealand

**Keywords:** beer, sound, esters, *Saccharomyces cerevisiae*, frequency, intensity, volatile organic compounds, fermentation

## Abstract

This study investigated the impact of varying sound conditions (frequency and intensity) on yeast growth, fermentation performance and production of volatile organic compounds (VOCs) in beer. Fermentations were carried out in plastic bags suspended in large water-filled containers fitted with underwater speakers. Ferments were subjected to either 200–800 or 800–2000 Hz at 124 and 140 dB @ 20 µPa. Headspace solid-phase microextraction (HS-SPME) coupled with gas chromatography-mass spectrometry (GC-MS) was used to identify and measure the relative abundance of the VOCs produced. Sound treatment had significant effects on the number of viable yeast cells in suspension at 10 and 24 h (*p* < 0.05), with control (silence) samples having the highest cell numbers. For wort gravity, there were significant differences between treatments at 24 and 48 h, with the silence control showing the lowest density before all ferments converged to the same final gravity at 140 h. A total of 33 VOCs were identified in the beer samples, including twelve esters, nine alcohols, three acids, three aldehydes, and six hop-derived compounds. Only the abundance of some alcohols showed any consistent response to the sound treatments. These results show that the application of audible sound via underwater transmission to a beer fermentation elicited limited changes to wort gravity and VOCs during fermentation.

## 1. Introduction

The demand for distinctly flavored beer is increasing; therefore, researchers are seeking reliable cost-efficient methods to enhance the aroma and flavor of beer and to optimize production. Fermentation by brewing yeast is responsible for the formation of important sensory characteristics in beer, including the production of volatile organic compounds (VOCs), such as higher alcohols, ‘fruity’ esters, vicinal diketones, and sulfur compounds. As VOC production is closely related to the growth and physiological state of the yeast, factors that affect yeast metabolism and physiology can impact on beer flavor [1]. As yeast also play a significant role in the biotransformation of hop-derived compounds to generate new VOCs, their growth and fermentation rate can be modified to optimize the production of hop derived VOCs [2]. Thus, any factors that affect yeast behavior have the potential to alter the production of VOCs.

Reports from previous studies suggest that audible sound stimulates the growth rate and production of metabolites in yeast. Collectively, the results discussed below suggest that applying sound to cultures of microorganisms, including yeast, may stimulate their growth and productivity. For instance, when the *Saccharomyces cerevisiae* strain VIN13 was cultured with sound stimulus, its growth rate (µ = 0.362 h^−1^) was 12.4% faster than in the control culture (µ = 0.322 h^−1^) [3]. Cells growing under low-frequency (100 Hz, 92 dB @ 20 μPa) and high-frequency (10 kHz, 89 dB @ 20 μPa) treatments have been reported to grow faster compared to cells growing in silence [3]. In addition, exposing *S*. *cerevisiae* C-2324 to low-power (0.3 W/L) ultrasound over 5 h increased their biomass concentration (from 0.12 to 0.4 g/L); however, increasing the power input to 12 W/L was not effective in enhancing either yeast growth or glucose utilization [4]. Subjecting *S*. *cerevisiae*-170 to the Hindustani classical music Ahir Bhairav raga (172–581 Hz, 70–90 dB @ 20 μPa) and Pilu raga (86–839 Hz, 85–110 dB @ 20 μPa) increased the yeast biomass concentration compared to a silence control [5]. A more recent study showed that audible sound stimulated the yeast growth rate by 23% compared to that of a silence control [6].

Other researchers have reported the effects of audible sound on bacteria. For example, subjecting *Brevibacterium* sp. to Tollywood music (100–1000 Hz, 60–90 dB @ 20 μPa) enhanced biomass and pigment production compared to the silence control [7]. Similarly, *Escherichia coli* K-12 grown under 8 KHz and 80 dB @ 20 μPa had a higher biomass (1.7 times) and a faster specific growth rate (2.5 times) compared to the control group (silence) [8].

From this prior research, it could be expected that applying sound to beer fermentation is likely to enhance yeast growth, thereby reducing fermentation and maturation time [3,6]. However, despite anecdotal accounts of the effect of sound on beer fermentation, research of this kind has not previously been reported. Therefore, this study was designed to assess the effects of sound frequency and intensity on yeast growth, fermentation, and the VOC composition of beer during fermentation using sound delivered via a water medium.

## 2. Results and Discussion

### 2.1. Yeast Number (Cells in Suspension)

Water was chosen as the sound transfer medium because sound waves experience substantial transmission loss and interference when passing between media of different densities, such as from air to liquid fermenting beer. In this regard, this study is thought to be the first report of an experiment where sound has been delivered via water to a liquid ferment during yeast fermentation.

**Figure 1:**
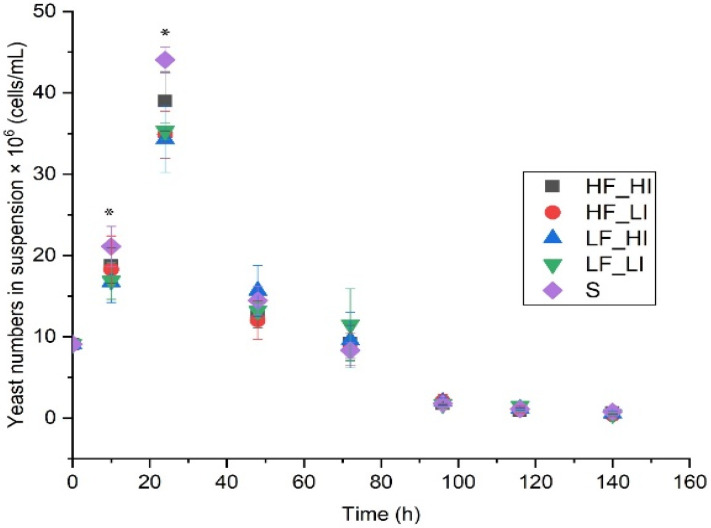
Viable yeast cells in suspension versus fermentation time for five sound treatments. Low-frequency and low intensity (LF_LI); low-frequency and high-intensity (LF_HI); high-frequency and low-intensity (HF_LI); high-frequency and high-intensity (HF_HI); silence (S). *Overall significant differences in means observed at 10 and 24 h (Table S1 Supplementary Material). Data shown are means of three fermentation replicates ± standard deviation (SD).

Yeast cells in suspension (viable cells) were measured at various time points during 144 h of fermentation (Figure 1). At time 0 h, the number of yeast cells was 9.1 ± 0.2 × 10^6^ cells/mL in all treatments, which was close to the target pitching rate of 10 million cells per mL. After 10 h of fermentation (post-pitching), the number of viable yeast cells in suspension increased to 16.8 ± 2.2 × 10^6^ cells/mL (low frequency, low intensity; LF_LI), 16.7 ± 2.4 × 10^6^ cells/mL (low frequency, high intensity; LF_HI), 18.3 ± 4.1 × 10^6^ cells/mL (high frequency_low intensity; HF_LI), 18.7 ± 2.1 × 10^6^ cells/mL (high frequency, high intensity; HF_HI), and 21.1 ± 2.4 × 10^6^ cells/mL (silence control; S) (Table S1 Supplementary Material). The treatment had a significant effect on viable yeast cells in suspension at 10 and 24 h (*p* < 0.05). Maximum yeast numbers occurred 24 h after fermentation commenced, with the silence group recording the highest number (44.5 ± 1.5 × 10^6^ cells/mL), which was significantly higher than LF_LI, LF_HI, and HF_LI but not different to HF_HI. The yeast numbers in suspension subsequently declined and converged with all other treatments at 48 h and for the rest of the fermentation to 144 h (Table S1 Supplementary Material).

The application of various sound intensities and frequencies has previously been shown to enhance the growth rates of *S*. *cerevisiae* [3,4,5,6,9], bacterial species [5,8,10,11,12,13], algae species [14,15], and plant species [16,17,18] compared to control experiments. However, in the current study, the growth rate was not calculated, and rather the number of yeast cells in suspension was reported. Therefore, our current findings are not directly comparable to previous studies. Sound treatments (Figure 1) did not enhance yeast numbers in suspension compared to the control (silence) group, which could potentially be ascribed to the following hypotheses: (1) stress, triggered as a result of sound exposure; (2) the specific frequency band delivered during fermentation did not have an effect on yeast in suspension; (3) the cell densities pitched might be too high to see an effect, thus inhibiting an increase in yeast growth as a function of the sound treatment; or (4) as yeast numbers in suspension were measured rather than optical density as in other studies, it is possible differences in total biomass were missed. Moreover, it has previously been reported that audible sound can exert inhibitory effects (osmotic stressors) on *E*. *coli*, thus decreasing growth and other biological activity [12].

### 2.2. Wort Gravity

**Figure 2:**
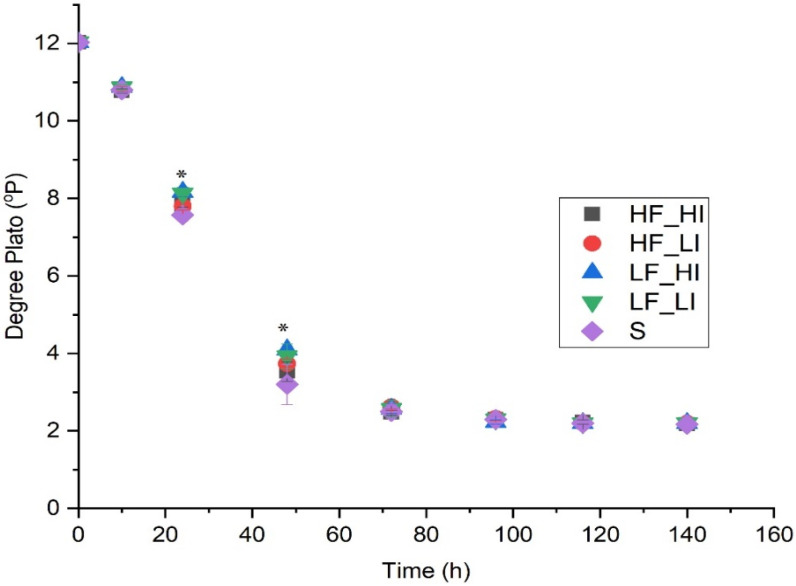
Wort gravity during fermentation over time for five sound treatments. Low-frequency and low-intensity (LF_LI); low-frequency and high-intensity (LF_HI); high-frequency and low-intensity (HF_LI); high-frequency and high-intensity (HF_HI); silence (S). * Significant differences observed at 24 and 48 h (Table S2 Supplementary Material). Data shown are the means of three fermentation replicates ± standard deviation.

A general decrease in wort gravity (measured according to density; °P) was observed for all treatments over the 144-h fermentation. There were significant differences among treatments at 24 and 48 h (Figure 2) before all treatments converged to the same gravity at 72 h, and reached final gravity (2.23 ± 0.06 °P) by 144 h. After 24 h, two treatments (LF_LI, LF_HI) showed higher gravity than the silence, namely HF_HI and HF_LI treatments (24 h, Table S2 Supplementary Material). After 48 h, samples from the silence group had the lowest gravity (3.20 ± 0.06 °P), which was significantly lower than the LF_HI treatment but not the remaining treatments.

Wort gravity (°P) represents the sugar content of the wort and decreases as fermentation progresses with conversion to metabolites and carbon dioxide (CO_2_) by the yeast. It is also used as a proxy to assess the fermentation performance of yeast [19,20,21,22]. It has previously been reported that the rate of sugar utilization by yeast in the presence of low energy ultrasound irradiation (20 kHz, 1 W/L) exposure was higher (98.9%) compared to the control group (92.4%) [9]. The application of various sounds has been reported to cause a more rapid decline in the density of the ferment compared to a silent control [5], which differs to the results from this current experiment. 

### 2.3. pH

**Figure 3:**
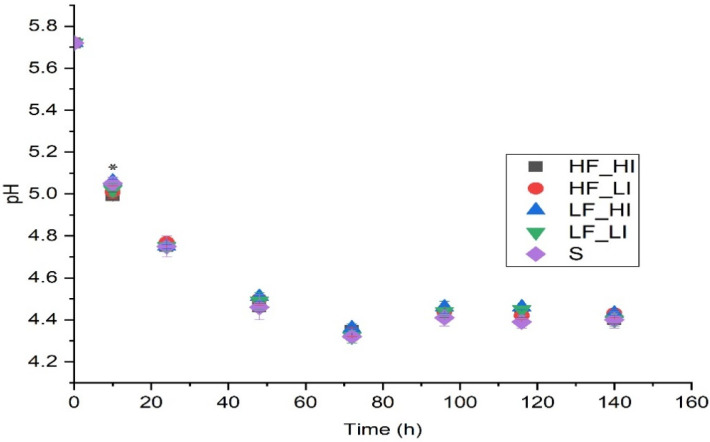
Change in pH during fermentation for five sound treatments. Low-frequency and low-intensity (LF_LI); low-frequency and high-intensity (LF_HI); high-frequency and low-intensity (HF_LI); high-frequency and high-intensity (HF_HI); silence (S). * Significant differences observed at 10 h. Results shown are means of three fermentation replicates ± standard deviation.

In the current study, pH decreased from 5.72 ± 0.01 to 4.40 ± 0.01 during fermentation, as expected [19] (Figure 3). There was only a statistical difference in pH between samples at 10 h for LF_HI and HF_HI, although the difference was very small (<0.1 pH unit). In beer production, pH is an essential factor because it influences yeast behavior and the synthesis of metabolites (alcohols, esters, etc.). 

### 2.4. Volatile Organic Compounds

A total of 33 volatile organic compounds (VOCs) were identified in the beer samples from the five sound treatments (Table A1). Among the VOCs identified, there were twelve esters, nine alcohols, three acids, three aldehydes, and six were hop-derived compounds. 

**Figure 4:**
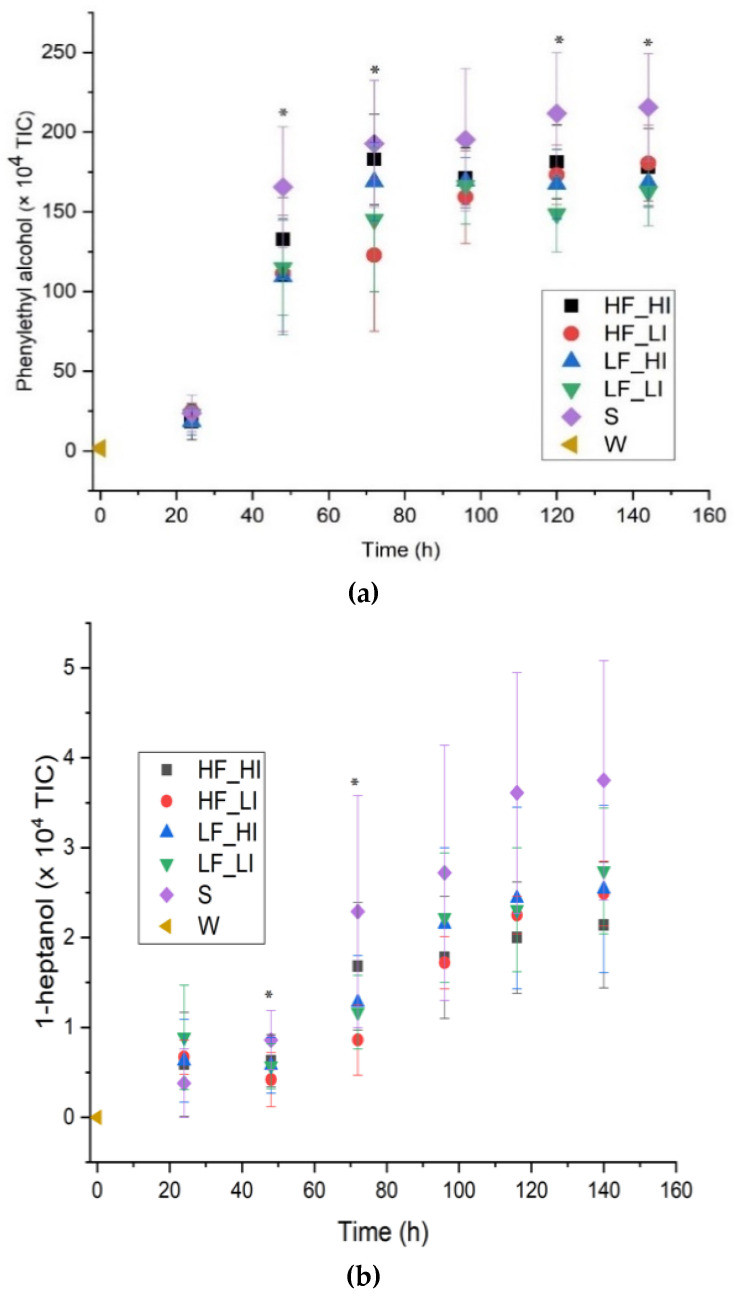
Abundance of higher alcohols during fermentation for five sound treatments including a silent control. (**a**) Phenylethyl alcohol (A32); (**b**) 1-heptanol (A15). Low-frequency and low-intensity (LF_LI); low-frequency and high-intensity (LF_HI); high-frequency and low-intensity (HF_LI); high-frequency and high-intensity (HF_HI); silence (S); wort (W). The results shown are means ± standard deviations of six measurements (3 biological fermentation replicates × 2 analytical replicates). * Significant difference observed (Table A1).

For the higher alcohols (HAs), the application of some of the sound treatments tended to decrease their synthesis, specifically for 2-methyl-1-propanol, 2-methyl-1-butanol, 3-methyl-1-butanol, 1-hexanol, and phenylethyl alcohol relative to the silence condition at each fermentation time point. For example, phenylethyl alcohol had a significantly higher (*p* < 0.05) abundance (higher peak area) in the silence control relative to treated samples at 48 (HF_LI, LF_HI, LF_LI), 72 (HF_LI, LF_LI), 120 (LF_LI), and 144 h (LF_HI, LF_LI) (Figure 4a; Table A1). These results suggest that sound treatment could potentially be utilized to reduce the concentration of phenylethyl alcohol. In addition, at 48 h, HF_LI had a significantly lower (*p* < 0.05) abundance of 1-heptanol (Figure 4b), relative to the silence control. Likewise, HF_LI-treated samples had a significant (*p* < 0.05) reduction in abundance of 1-heptanol at 72 h. At 144 h, HF_LI and the silence control had a higher abundance of 2-methyl-1-butanol compared to the rest of the treatments (i.e., HF_HI, LF_HF, LF_LI). However, the abundance of 2-methyl-1-butanol and 1-hexanol converged at the end of the fermentation (144 h), thus resulting in no differences in their abundance being detected in the final beer (*p* > 0.05). It has been previously reported that the predominant HAs in beer are 3-methyl-1-butanol (60–80%), 2-methyl-1-propanol (15–25%), and 1-propanol (4–7%), which are formed as byproducts during biosynthesis of amino acids [23,24,25]. Amino acid biosynthesis has previously been reported to be upregulated as a result of sound treatments (music and low-frequency sound (100 Hz, 92 dB @ 20 µPa) [3]. The decrease in HAs synthesis at certain sampling times during fermentation observed in this study may be due to inhibition of aminotransferases, pyruvate decarboxylases (*pdc1*, *pdc5*, and *pdc6*), and alcohol dehydrogenases (*Adh1*, *Adh2*, *Adh3*, *Adh4*, and *Adh5* or *Sfa1*) [26,27].

**Figure 5:**
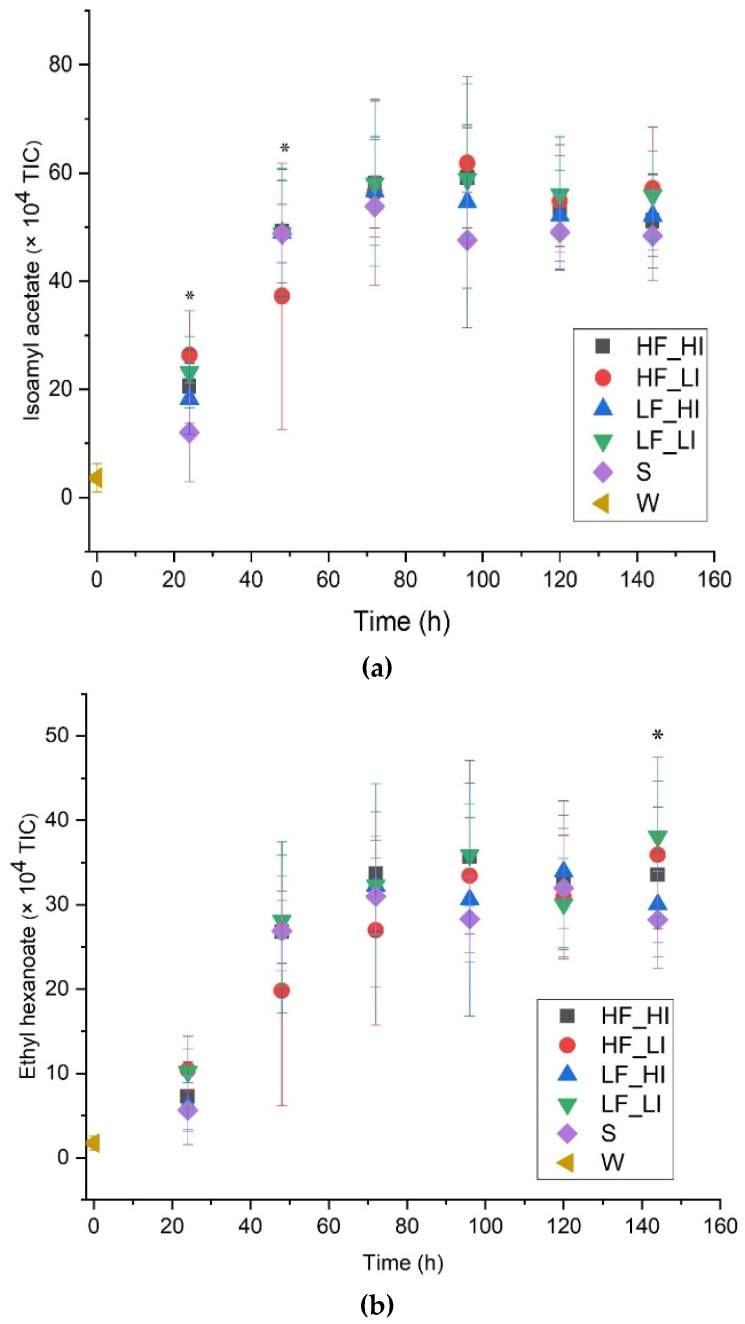
Abundance of esters during fermentation for five sound treatments, including a silent control. (**a**) Isoamyl acetate (A6); (**b**) ethyl hexanoate (A10). Low-frequency and low-intensity (LF_LI); low-frequency and high-intensity (LF_HI); high-frequency and low-intensity (HF_LI); high-frequency and high-intensity (HF_HI); silence (S); wort (W). The results shown are means ± standard deviations of six measurements (3 biological fermentation replicates × 2 analytical replicates). Total Ion Chromatogram (TIC). * Significant difference observed among treatments (Table A1).

Despite the yeast growth and fermentation rate not being significantly altered, subtle differences for some yeast-derived esters were observed at certain times during fermentation. The abundance for isoamyl acetate (Figure 5a) at 24 h for HF_LI was significantly higher (*p* < 0.05) than for silence by 14%. At 144 h, significant differences in abundance between some treatments for ethyl hexanoate, ethyl octanoate, and phenylethyl acetate were observed. Specifically, the abundance for ethyl hexanoate was significantly (*p* = 0.001) higher for the LF_LI-treated ferment compared to the silence control but not for the rest of the treatments at 144 h (Figure 5b). HF_HI, LF_LI, and HF_LI exhibited a higher abundance of ethyl octanoate compared to LF_HI and silence at 144 h. For phenethyl acetate, its abundance in the HF_LI-treated ferment was significantly higher (*p* < 0.05) than for LF_HI at 144 h but not HF_HI, LF_LI, or silence. Despite these differences, there were little consistent effects of sound treatment on the abundance of esters in the experimental samples under the current conditions.

Volatile esters impart beer with fruity, candy, and perfume-like flavor characters [28,29]. Acyl-coenzyme A and acetyltransferase catalyze the synthesis of esters. It has previously been reported that ultrasound and cavitational implosion can alter monomeric and polymeric enzymes in yeast [4]. Therefore, the difference observed in the abundance of isoamyl acetate and ethyl hexanoate may be due to differences in acetyltransferase enzymatic activity triggered by the sound treatments. However, it has also been reported that hydrostatic pressure, and the amount of nitrogen and glucose in wort can alter ester synthesis [30,31]. The application of high (10 kHz, 90 dB @ 20 µPa) and low (100 Hz, 90 dB @ 20 µPa) audible sound to yeast significantly decreased the production of ethyl octanoate compared to the silence control [6]. Therefore, it is also possible that the changes in cell numbers in suspension and/or glucose utilization may underlie the differences in the observed levels of esters in our ferments.

The three organic acids identified in the current study, 2-methylpropanoic acid, hexanoic acid, and octanoic acid, showed no significant (*p* > 0.05) effects on their abundance during fermentation (Table A1) as a result of sound treatment. The lack of a significant impact of sound on organic acid production does not appear to result from an inability to detect organic acids, as increases in the abundance of hexanoic acid were detected over the course of the fermentation. 

**Figure 6:**
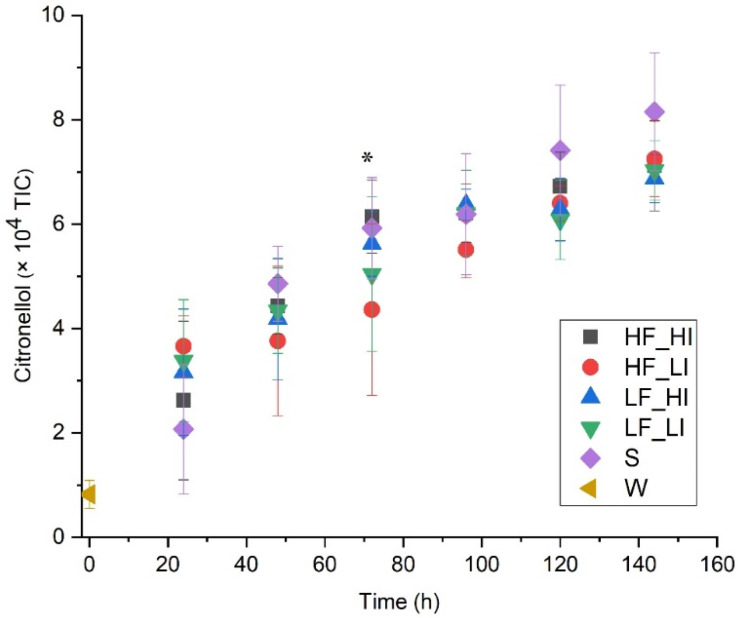
Abundance of Citronellol (A25) during fermentation for five sound treatments including a silent control. Low-frequency and low-intensity (LF_LI); low-frequency and high-intensity (LF_HI); high-frequency and low-intensity (HF_LI); high-frequency and high-intensity (HF_HI); silence (S); wort (W). The results shown are means ± standard deviations of 6 measurements (3 biological fermentation replicates × 2 analytical replicates). * Significant difference observed among treatments (see Table A1).

Citronellol, linalool, 1,2-dihydrolinalool, geraniol, and 2-methylbutyl isobutyrate are hop-derived compounds that were identified in the present study. Citronellol (Figure 6) increased over time in all treatment samples, likely due to yeast biotransformation reactions, as previously reported [32,33], as did 1,2-dihydrolinalool (Table A1). It has previously been reported that higher audible sound (10 kHz, 90 dB @ 20 µPa) enhanced (by 7.8-fold) the production of limonene by yeast compared to the silence control [6]. In contrast, the abundance of 2-methylbutyl isobutyrate, linalool, and geraniol decreased as fermentation progressed in this study. The decreases in the abundance of these hop-derived compounds may result from stripping effects of CO_2_ during fermentation or from biotransformation reactions by yeast [32,33]. However, no consistent effects of sound treatment were observed for any of these hop-derived compounds.

**Figure 7:**
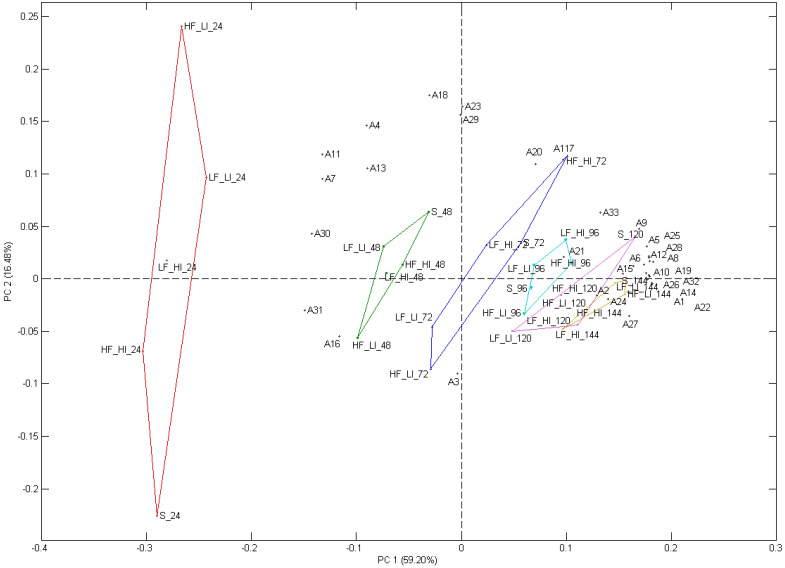
Biplots of the principal component analysis of the VOCs for sequential time intervals in beer brewed under five different sound treatments including a silent control. The numbers representing the volatile organic compounds matches the numbers used in Table A1. Low-frequency and low-intensity (LF_LI); low-frequency and high-intensity (LF_HI); high-frequency and low-intensity (HF_LI); high-frequency and high-intensity (HF_HI), (S) Silence–control.

Principal component analysis (PCA) was performed to visualize the relationships between treatments, fermentation time and the VOCs identified (Figure 7). The first two principal components, PC1 and PC2, accounted for 75.68% of the total variability. Fermentation time (h) dominated the explained variance and explained the separation on PC1, where ferments at 24 h (on left) were separated from ferments at 144 and 120 h (on right), specifically S_120, S_144, HF_LI_144, and LF_LI_144. On PC1, the majority of VOCs had high positive loadings and contributed more to the separation of samples on PC1 than the VOC with negative loadings. The compounds that were most positively associated with fermentation time at S_120, S_144, HF_LI_144, and LF_LI_144 on PC1 were 3-methyl butyl octanoate, phenylethyl acetate, ethyl acetate, ethyl octanoate, ethyl 9-decenoate, ethyl, and 1-heptanol. Some yeast metabolites are produced and accumulate during fermentation. Samples at 24 and 48 h (connected by red and green lines) were associated with a lower abundance of the above compounds and positively associated with a higher abundance of 2,2,4-trimethyl-1,3-pentanediol isobutyrate, ethyl 1-hexanol, ethyl dodecanoate, 2-methylbutyl isobutyrate, and methyl 4-methylenehexanoate. Separation on PC2 was primarily due to HF_LI and silence conditions at 24 h of fermentation time. This separation was largely related to the positive loadings of linalool, the unknown terpene alcohol (A23, 21.18 min), 2-methylbutyl isobutyrate, and geraniol, which indicates higher levels of these compounds in HF_LI treatment at 24 h. A decrease in hop-derived VOCs was also observed as fermentation time increased (Table A1).

## 3. Materials and Methods

### 3.1. Materials and Chemicals

Spray-dried malt extract (Briess Industries, Inc., USA) and calcium chloride (CaCl) were obtained from a local supplier (www.brewshop.co.nz, accessed on 3 November 2021; Hamilton, New Zealand). Sodium chloride (NaCl, analytical grade) was purchased from Merck (Darmstadt, Hessen, Germany). T90 hop pellets of the cultivars Waimea (bittering hop) and Motueka (aroma hops) were supplied by NZ Hops Limited (Tasman, New Zealand). *Saccharomyces cerevisiae* Safale US-05 was provided by Fermentis (Lille, France). Yeast cell counts were conducted using a Oculyze BB 1.0 microscope (Oculyze GmbH, Hochschulring, Germany), consisting of a 200 µL sample chamber (Gräfelfing, Germany) and an LG smartphone device (LG Electronics, South Korea). Reinforced nylon EVOH/LLDPE wine bags (3 L; DS Smith Plc, London, UK), used to conduct fermentations in, were obtained from DS Smith (Auckland, New Zealand). Large 115 L polyethylene tanks (D115 container with lid (3660PL; Stowers Containment Solutions, Christchurch, NZ) were used to house the underwater sound experiments. 

### 3.2. Yeast Activation

Malt extract (127 g) was dissolved in 1000 mL of tap water in a conical flask to achieve 12°P. A magnetic stirbar was dropped into the solution and the solution was autoclaved at 120 °C for 15 min. The wort solution was cooled to 20 °C prior to inoculation. Dry yeast (US-05, 11 g) was weighed, pitched, and the flask capped with an airlock. The flask was incubated at 20 °C with continuous stirring for 24 h. The slurry of propagated yeast cells and media was centrifuged (3000 rpm for 10 min) and the supernatant discarded. The yeast slurry was resuspended in fresh wort (1 L) and vortexed prior to pitching. 

#### 3.2.1. Yeast Quantification and Pitching

Yeast cell numbers were determined using Oculyze BB 1.0 with methylene blue as a stain. The number of the viable yeast cells were calculated by pipetting 1 mL of the slurry into 99 mL of water. The diluted slurry (1 mL) was mixed with methylene blue stain (1:1 ratio) and allowed to rest for 30 s in a 2 mL microcentrifuge tube. The sample was then loaded into the chamber of an Oculyze-microscope slide using a micropipette. The yeast count (million cells/mL viable cells) was determined using five images [34]. The volume of yeast slurry required to achieve a standard pitching rate (1.0 × 10^7^ cells/mL) was calculated for inoculation of the fermentation samples. 

### 3.3. Preparation of Wort

Malt extract (1.44 kg) was used to prepare the wort using filtered water (municipal supply; 12 L) for the mixing and adjustment of the density (°P). The wort was boiled for 30 min. Once boiling started, CaCl (0.996 g; to achieve 50 ppm) and Waimea bittering hop was added to achieve a standardized bitterness (~25 International Bittering Units (IBU)). Before cooling, Motueka hops (5 g/L) were added, and the temperature kept at 90 °C for 5 min. Cooling of the wort to ~20 °C was accomplished with the aid of a sterilized immersion wort chiller, which was immersed in the wort before boiling commenced (30 min). The cooled wort (12 L) was aerated with the aid of an aeration stone (pore size: 0.5 µm) and membrane air pump (10 min, 8 psi). The ferments were bulk pitched to ensure that the inoculation rate was identical, and the pitched wort was distributed into individual wine fermentation bags. The bags containing the pitched wort were heat sealed using an impulse heat sealer prior to fermentation (Section 3.4.1, Table 1).

### 3.4. Sound Generation

Sound files (3 min duration) were generated at different frequency ranges (Table 1; Audio S1) with bespoke MATLAB^®^ (Version R2019a; Math Works, MA, USA) scripts (Supplementary Data S1) and stored as WAV files. The files were burned on a compact disc and played continuously with Groov-e GVPS110SR retro series CD Players (Groov-e^®^, China). The sound signals were amplified by 1000 W power amplifiers (Pioneer Gm-A6704 A Series, Japan) connected to power adapters, CD Players, and underwater speakers (LL916C-050, Lubell Labs Inc., USA). The volume functions of the CD Players and the amplifiers were used to adjust the sound delivered to achieve the desired sound intensity levels.

#### 3.4.1. Sound Delivery and Fermentation

**Figure 8:**
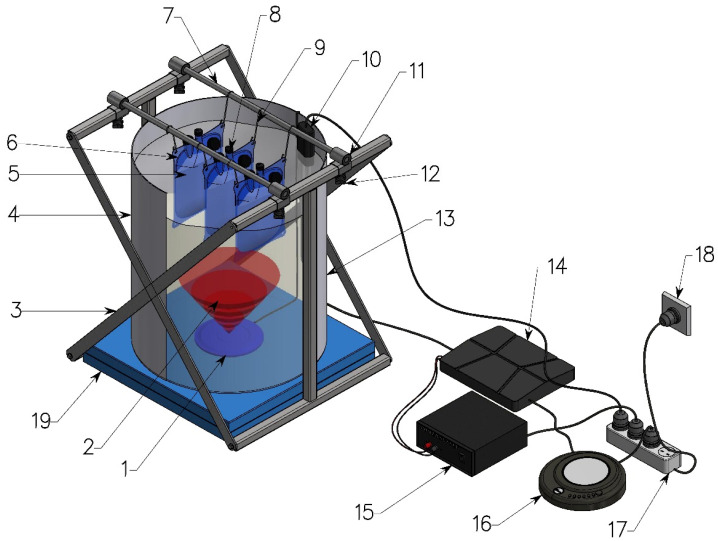
Experimental setup. 1. underwater speaker; 2. sound travelling via water; 3. support stand; 4. water level; 5. pitched wort; 6. submerged fabricated wine bag; 7. cross beam; 8. an airlock; 9. rubber band attached to hook for acoustically isolating suspended wine bag; 10. aquatic water heater; 11. foam tube; 12. adjustable knob; 13. plastic cylindrical container; 14. 1000 W power amplifier; 15. power adapter; 16. CD player; 17. multiple outlet extension cord; 18. wall socket; 19. foam pads for sound proofing.

Large water-filled vessels (D115 containers) were used provide a liquid medium to transmit the sound using an underwater speaker positioned in the bottom of the tank, with submerged fermentation bags suspended in the water above (Figure 8). The vessels were placed on sound-suppressing foam pads to reduce sound and vibration transfer among experimental units. Fermentation was carried out at 20 °C until a consistent gravity reading was achieved for all samples. Before commencement of the fermentation, a calibrated HTI-96-Min broadband hydrophone (High Tech Inc., Long Beach, MS, USA) with a flat frequency response over the audible frequency range was used to quantify the background noise in each vessel and to adjust the intensity of the underwater sound for the sound treatments to the required level. A period of the outputs (10 s) was recorded using a digital recorder (R-05 Recorder, Roland Corporation, Japan) and analyzed in MATLAB^®^ with different bespoke scripts (Supplementary Data S2) to calculate the mean sound intensity and frequency composition of each recording (Figure S1 Supplementary Material). 

Each treatment was run in triplicate (3 separate fermentation bags) to obtain a measure of biological variation. Samples (50 mL) were withdrawn with the aid of a sterilized pipette at particular time-points (0, 10, 24, 48, 72, 96, 120, 144 h) to monitor fermentation performance and for VOC analysis. Samples for VOC analysis were transferred immediately after sampling into 50 mL Falcon tubes and centrifuged at 3000 rpm for 15 min. The supernatant (beer) was added to fresh Falcon tubes, capped, and frozen. Centrifugation was carried out to remove all suspended yeast, thus avoiding yeast autolysis, which may have altered the VOCs present in the stored samples. 

### 3.5. Physicochemical Parameters

The apparent extract (°P) of the wort (gravity) during fermentation was determined using a handheld density meter (Anton Paar, Austria). The pH was determined using a digital pH meter (Ohaus^®^, China). Before any of the analysis mentioned above, beer samples were degassed by sonication.

#### Yeast in Suspension and Viability 

Yeast numbers in suspension (viable cells) were estimated at 0, 10, 24, 24, 48, 72, 96, 120, and 144 h over the course of the fermentation for the five treatment conditions using the protocol described above (Section 3.2.1).

### 3.6. VOC Analysis

Headspace solid-phase microextraction (HS-SPME) coupled with gas chromatography-mass spectrometry (GC-MS) was used to identify and measure the relative abundance of the VOCs in the beer samples according to a method described previously, with some modifications [35]. Frozen beer samples were thawed and 8 mL of sample introduced into a 20 mL headspace vial containing NaCl (2.5 g). The vials were tightly sealed with PTFE-coated silicone septa and incubated for 3 min at 40 °C in a thermostatic agitator. The extractions were carried out with a multipurpose autosampler (MPS, Gerstel) for 30 min using a divinylbenzene/carboxen/polydimethylsiloxane (DVB/CAR/PDMS) coated fiber (1 cm, 40 μm) in static headspace mode. The compounds were thermally desorbed at 240 °C for 5 min in splitless mode (GC split/splitless inlet, Agilent) with a purge flow of 60 mL min after 2 min.

#### 3.6.1. GC–MS Conditions

The VOCs were analyzed using an Agilent 7890B GC coupled to an Agilent MSD 5977A quadrupole mass spectrometer (Agilent Technologies, Beijing, China). The chromatographic separation was carried out with 30 m × 0.25 mm I.D. × 0.25 μm SOLGEL-WAX column (SGE Analytical Science, Ringwood, Australia) with hydrogen as the carrier gas at a flow rate of 1.6 mL/min. The initial pressure was 4.6 psi with the average velocity being 26 cm/s. The oven temperature was held at 40 °C for 3 min, increased at a rate of 3 °C/min to 100 °C, then increased at 4 °C/min to 250 °C, and held for 5 min. The transfer line to the MS and the quadrupole were set to 230 and 150 °C, respectively. The mass spectrometer was operated at a scan speed of 5.1 scans/s and mass spectra recorded in the range of 30–300 m/z. Carryover between GC runs was evaluated using empty vials as blanks before and after every 31 samples.

#### 3.6.2. Data Analysis

Tentative identification of the VOCs was performed by PARAllel FACtor analysis 2 (PARAFAC2) based Deconvolution and Identification System (PARADISe) software [36]. VOCs were identified by comparing the deconvoluted mass spectra of each compound in the National Institute of Standard and Technology library (NIST 2014).

### 3.7. Statistical Analysis

Data generated during fermentation were conducted in three fermentation replicates (i.e., separate bags), and the results reported as mean ± standard deviation (SD). VOCs data were analyzed with six replicates (3 fermentation replicates × 2 analytical replicates). A general linear model was used to identify the significant (*p* < 0.05) treatment effects for each variable. Where significant overall effects were identified, it was followed by a pairwise comparison of means using a Tukey’s test. All statistical analyses and figures were performed using Minitab^®^ 18 (Minitab, LLC, PA, USA) and OriginPro (OriginLab, Massachusetts, USA), respectively. Principal component analysis was carried out using the mean averages of the VOCs for each sound treatment at each fermentation time point in Solo (Version 6.5, 2018, Eigenvector Research, Wenatchee, WA, USA).

## 4. Conclusions

In a closely controlled experiment, the underwater application of audible sound to beer fermentations elicited limited changes to the number of yeast cells in suspension, wort gravity, or the composition and abundance of VOCs. These results contrast with those generally reported that typically observe significantly enhanced yeast growth and metabolite production. Therefore, further investigation is required to determine whether the different sound delivery parameters employed in the current study underlie why differences mediated by audible sound were not observed.

## Figures and Tables

**Table 1:** Experimental design.

Barrel	Frequency (Hz)	Intensity (dB)	Temperature (°C)	Treatment
1	200–800	124 ^†^	20	Low frequency_low intensity (LF_LI)
2	800–2000	140 ^†^	20	High frequency_ High intensity (HF_HI)
3	800–2000	124 ^†^	20	High frequency_low intensity (HF_LI)
4	200–800	140 ^†^	20	Low frequency_high intensity (LF_HI)
5	Silence	94.55 ^β^	20	Silence (S)

Three fermentation replicates each, summing up to a total of 15 experimental fermentation units. ^†^ and ^β^ were measured at 20% and 80% hydrophone levels, respectively.

## Data Availability

Not applicable.

## Supplementary Materials

The following are available online, Table S1. Viable yeast numbers in suspension (×10^6^ cells/mL) during fermentation for five treatment conditions; Table S2. Wort gravity (°P) during fermentation over time for five treatment conditions; Supplementary Data S1. Bespoke MATLAB^®^ scripts for sound generation; Audio S1. Sound generated by Bespoke MATLAB^®^ scripts (Supplementary Data S1) and saved as WAV file (a. 200–800 Hz; b. 800–2000 Hz); Supplementary Data S2. Bespoke MATLAB^®^ scripts for calculating the mean level of sound intensity delivered before commencing fermentation; Figure S1. The mean intensity levels measured for baseline (background noise 94.55 dB @ 1 µPa (a); 124.03 dB @ 20 µPa at 124 Hz (b); 140.01 dB @ 20 µPa at 800–2000 Hz (c).

## Appendix A

**Table A1:** Peak response areas (×10^4^ TIC) of volatile organic compounds detected in beer fermentation under five sound treatments at 24, 48, 73, 96, 120, and 144 h sampling intervals during fermentation.

**24 h**								
**Codes**	**Volatile organic compounds**	**RT**	**HF_HI**	**HF_LI**	**LF_HI**	**LF_LI**	**S**	***p* value**
A1	Ethyl acetate	2.43	8.21 ± 2.89 ^a^	8.61 ± 2.23 ^a^	8.49 ± 2.52 ^a^	9.49 ± 3.29 ^a^	5.51 ± 4.12 ^a^	0.206
A2	1,1-diethoxyethane	2.5	0.35 ± 0.23 ^a^	0.52 ± 0.04 ^a^	0.31 ± 0.28 ^a^	0.64 ± 0.22 ^a^	0.34 ± 0.35 ^a^	0.147
A3	Ethanol	3.08	1124.33 ± 322.34 ^ab^	753.61 ± 338.25 ^b^	1151.64 ± 323.96 ^ab^	1187.76 ± 193.40 ^ab^	1338.22 ± 275.64 ^a^	**0.058**
A4	2,6-dimethyl-2-trans-6-octadiene	6	0.40 ± 0.17 ^ab^	0.54 ± 0.12 ^a^	0.42 ± 0.13 ^ab^	0.41 ± 0.12 ^ab^	0.24 ± 0.13 ^b^	**0.03**
A5	2-methyl-1-propanol	6.12	10.11 ± 5.25 ^a^	12.03 ± 2.93 ^a^	12.06 ± 4.46 ^a^	13.04 ± 5.45 ^a^	10.02 ± 5.32 ^a^	0.801
A6	Isoamyl acetate	6.76	20.53 ± 6.78 ^ab^	26.31 ± 8.19 ^a^	18.24 ± 6.54 ^ab^	23.17 ± 6.60 ^ab^	12.03 ± 9.19 ^b^	**0.032**
A7	2-methylbutyl isobutyrate	8.68	1.59 ± 1.13 ^ab^	3.39 ± 2.18 ^a^	0.85 ± 0.58 ^b^	1.67 ± 1.30 ^ab^	0.51 ± 0.44 ^b^	**0.012**
A8	2-methyl-1-butanol	9.18	39.95 ± 17.89 ^a^	47.48 ± 8.18 ^a^	44.57 ± 14.50 ^a^	48.80 ± 17.98 ^a^	38.72 ± 18.95 ^a^	0.809
A9	3-methyl-1-butanol	9.23	104.24 ± 46.43 ^a^	123.67 ± 20.84 ^a^	115.67 ± 37.05 ^a^	126.23 ± 45.09 ^a^	99.72 ± 49.00 ^a^	0.977
A10	Ethyl hexanoate	9.84	7.26 ± 4.11 ^a^	10.38 ± 2.54 ^a^	6.12 ± 2.79 ^a^	10.16 ± 4.24 ^a^	5.62 ± 4.07 ^a^	0.087
A11	Methyl 4-methylenehexanoate	12.42	1.11 ± 0.97 ^a^	1.89 ± 0.93 ^a^	1.16 ± 0.92 ^a^	1.59 ± 0.80 ^a^	0.47 ± 0.58 ^b^	**0.052**
A12	Ethyl heptanoate	12.53	0.41 ± 0.28 ^a^	0.49 ± 0.23 ^a^	0.31 ± 0.22 ^a^	0.58 ± 0.40 ^a^	0.32 ± 0.34 ^a^	0.523
A13	1-hexanol	12.95	0.74 ± 0.45 ^a^	0.96 ± 0.19 ^a^	0.92 ± 0.42 ^a^	0.95 ± 0.38 ^a^	0.53 ± 0.40 ^a^	0.33
A14	Ethyl octanoate	15.34	28.86 ± 17.30 ^a^	39.05 ± 9.82 ^a^	23.38 ± 8.03 ^a^	38.93 ± 20.96 ^a^	27.12 ± 16.38 ^a^	0.363
A15	1-heptanol	15.58	0.59 ± 0.58 ^a^	0.67 ± 0.19 ^a^	0.63 ± 0.46 ^a^	0.89 ± 0.58 ^a^	0.38 ± 0.38 ^a^	0.506
A16	Ethyl-1-hexanol	16.99	1.21 ± 1.05 ^a^	0.40 ± 0.18 ^a^	1.28 ± 1.20 ^a^	0.77 ± 0.62 ^a^	1.64 ± 1.88 ^a^	0.418
A17	1,2-dihydrolinalool	17.55	5.51 ± 2.72 ^a^	6.17 ± 0.44 ^a^	6.38 ± 2.67 ^a^	7.05 ± 2.78 ^a^	3.96 ± 3.02 ^a^	0.384
A18	Linalool	17.85	30.26 ± 18.74 ^a^	45.56 ± 6.44 ^a^	37.50 ± 16.44 ^a^	37.82 ± 12.67 ^a^	20.63 ± 17.66 ^b^	**0.047**
A19	1-octanol	18.1	1.21 ± 0.60 ^a^	1.64 ± 0.18 ^a^	1.44 ± 0.46 ^a^	1.45 ± 0.53 ^a^	0.96 ± 0.50 ^a^	0.257
A20	2-methylpropanoic acid	18.16	1.31 ± 0.87 ^a^	1.91 ± 0.39 ^a^	1.60 ± 0.68 ^a^	1.86 ± 0.72 ^a^	1.66 ± 0.78 ^a^	0.725
A21	Ethyl decanoate	20.14	12.01 ± 6.17 ^a^	16.86 ± 4.14 ^a^	10.10 ± 3.63 ^a^	19.67 ± 10.35 ^a^	13.95 ± 8.15 ^a^	0.256
A22	3-methylbutyl octanoate	20.59	0.85 ± 0.48 ^a^	0.97 ± 0.23 ^a^	0.67 ± 0.18 ^a^	1.18 ± 0.75 ^a^	0.95 ± 0.57 ^a^	0.565
A23	Unknown terpene alcohol	21.18	1.22 ± 1.13 ^a^	2.16 ± 0.26 ^a^	1.50 ± 1.10 ^a^	1.74 ± 0.88 ^a^	0.94 ± 0.89 ^a^	0.332
A24	Ethyl 9-decenoate	21.23	0.22 ± 0.27 ^a^	ND	ND	1.01 ± 1.96 ^a^	0.44 ± 0.65 ^a^	0.418
A25	Citronellol	22.76	2.62 ± 1.52 ^a^	3.66 ± 0.58 ^a^	3.16 ± 1.21 ^a^	3.38 ± 1.17 ^a^	2.07 ± 1.24 ^a^	0.272
A26	3,5-dimethyl-benzaldehyde	23.51	0.71± 0.44 ^a^	0.54 ± 0.18 ^a^	1.02 ± 0.76 ^a^	0.57 ± 0.33 ^a^	0.84 ± 0.83 ^a^	0.556
A27	Phenethyl acetate	23.65	1.58 ± 0.88 ^a^	2.17 ± 0.34 ^a^	1.67 ± 0.67 ^a^	1.98 ± 0.68 ^a^	1.51 ± 0.81 ^a^	0.567
A28	Hexanoic acid	24.26	3.49 ± 2.77 ^a^	5.04 ± 0.99 ^a^	3.82 ± 2.14 ^a^	4.80 ± 2.18 ^a^	3.50 ± 2.40 ^a^	0.709
A29	Geraniol	24.42	3.52 ± 2.93 ^a^	5.69 ± 1.04 ^a^	4.81 ± 3.16 ^a^	4.88 ± 2.27 ^a^	2.73 ± 2.46 ^a^	0.413
A30	Ethyl dodecanoate	24.57	4.29 ± 1.44 ^a^	6.49 ± 1.42 ^a^	4.21 ± 1.40 ^a^	4.80 ± 2.24 ^a^	3.89 ± 2.28 ^a^	0.229
A31	2,2,4-trimethyl-1,3-pentanediol isobutyrate	25.12	8.55 ± 6.20 ^a^	4.31 ± 1.13 ^a^	6.89 ± 4.86 ^a^	6.73 ± 3.58 ^a^	7.28 ± 5.24 ^a^	0.981
A32	Phenylethyl alcohol	25.57	18.12 ± 11.11 ^a^	24.80 ± 3.47 ^a^	18.40 ± 8.35 ^a^	22.78 ± 7.28 ^a^	23.68 ± 11.54 ^a^	0.662
A33	Octanoic acid	28.52	30.33 ± 25.29 ^a^	48.68 ± 10.53 ^a^	35.43 ± 21.99 ^a^	45.56 ± 20.88 ^a^	29.82 ± 24.35 ^a^	0.56
**48 h**								
**Codes**	**Volatile organic compounds**	**RT**	**HF_HI**	**HF_LI**	**LF_HI**	**LF_LI**	**S**	***p* value**
A1	Ethyl acetate	2.43	18.67 ± 4.96 ^a^	14.77 ± 9.21 ^a^	18.69 ± 4.96 ^a^	17.26 ± 4.29 ^a^	18.71 ± 2.14 ^a^	0.663
A2	1,1-diethoxyethane	2.5	0.21 ± 0.13 ^b^	0.52 ± 0.36 ^ab^	0.74 ± 0.39 ^a^	0.71 ± 0.16 ^ab^	0.65 ± 0.22 ^ab^	**0.05**
A3	Ethanol	3.08	1279.77 ± 522.72 ^a^	1016.36 ± 137.43 ^a^	1230.44 ± 163.86 ^a^	1254.08 ± 160.52 ^a^	1250.48 ± 210.65 ^a^	0.537
A4	2,6-dimethyl-2-trans-6-octadiene	6	0.37 ± 0.03 ^ab^	0.29 ± 0.11 ^b^	0.39 ± 0.09 ^ab^	0.41 ± 0.09 ^a^	0.39 ± 0.06 ^ab^	**0.042**
A5	2-methyl-1-propanol	6.12	14.26 ± 4.47 ^a^	14.59 ± 5.67 ^a^	14.42 ± 5.33 ^a^	13.56 ± 3.69 ^a^	17.40 ± 2.56 ^a^	0.241
A6	Isoamyl acetate	6.76	49.13 ± 9.47 ^a^	37.21 ± 24.64 ^b^	48.89 ± 11.77 ^a^	48.77 ± 12.11 ^a^	48.78 ± 5.41 ^a^	**0.051**
A7	2-methylbutyl isobutyrate	8.68	0.76 ± 0.73 ^a^	0.66 ± 0.78 ^a^	0.48 ± 0.34 ^a^	0.82 ± 0.37 ^a^	0.58 ± 0.76 ^a^	0.896
A8	2-methyl-1-butanol	9.18	81.53 ± 18.78 ^a^	73.98 ± 25.60 ^a^	75.84 ± 22.11 ^a^	73.33 ± 15.24 ^a^	88.07 ± 10.13 ^a^	0.26
A9	3-methyl-1-butanol	9.23	159.77 ± 35.26 ^a^	149.23 ± 51.22 ^a^	153.71 ± 44.77 ^a^	148.94 ± 31.24 ^a^	168.93 ± 16.98 ^a^	0.56
A10	Ethyl hexanoate	9.84	26.79 ± 3.76 ^a^	19.79 ± 13.59 ^a^	27.32 ± 10.16 ^a^	28.12 ± 7.76 ^a^	26.87 ± 4.74 ^a^	0.447
A11	Methyl 4-methylenehexanoate	12.42	0.76 ± 0.20 ^a^	0.59 ± 0.56 ^a^	0.83 ± 0.44 ^a^	0.88 ± 0.44 ^a^	0.73 ± 0.26 ^a^	0.64
A12	Ethyl heptanoate	12.53	0.90 ± 0.40 ^a^	0.57 ± 0.69 ^a^	1.05 ± 0.57 ^a^	1.08 ± 0.50 ^a^	0.96 ± 0.25 ^a^	0.434
A13	1-hexanol	12.95	0.60 ± 0.15 ^a^	0.49 ± 0.24 ^a^	0.54 ± 0.19 ^a^	0.52 ± 0.16 ^a^	0.58 ± 0.08 ^a^	0.269
A14	Ethyl octanoate	15.34	97.17 ± 36.89 ^a^	93.38 ± 55.96 ^a^	113.34 ± 59.86 ^a^	120.41 ± 52.76 ^a^	107.98 ± 17.94 ^a^	0.785
A15	1-heptanol	15.85	0.63 ± 0.29 ^ab^	0.42 ± 0.30 ^b^	0.58 ± 0.31 ^ab^	0.57 ± 0.25 ^ab^	0.86 ± 0.33 ^a^	**0.052**
A16	Ethyl-1-hexanol	16.99	1.03 ± 0.26 ^a^	1.44 ± 1.41 ^a^	1.13 ± 0.33 ^a^	1.96 ± 2.73 ^a^	0.99 ± 0.23 ^a^	0.7
A17	1,2-dihydrolinalool	17.55	6.20 ± 1.66 ^a^	5.08 ± 2.01 ^a^	5.96 ± 1.43 ^a^	5.94 ± 1.12 ^a^	5.81 ± 1.16 ^a^	0.676
A18	Linalool	17.85	33.14 ± 2.69 ^a^	30.48 ± 12.34 ^a^	36.21 ± 10.16 ^a^	36.21 ± 6.51 ^a^	36.05 ± 3.62 ^a^	0.584
A19	1-octanol	18.1	2.80 ± 0.56 ^a^	2.31 ± 0.89 ^a^	2.83 ± 0.90 ^a^	2.85 ± 0.71 ^a^	2.97 ± 0.41 ^a^	0.41
A20	2-methylpropanoic acid	18.16	1.56 ± 0.33 ^a^	1.66 ± 0.69 ^a^	1.58 ± 0.52 ^a^	1.61 ± 0.36 ^a^	1.81 ± 0.26 ^a^	0.655
A21	Ethyl decanoate	20.14	32.98 ± 15.17 ^a^	33.90 ± 17.01 ^a^	42.59 ± 23.22 ^a^	47.79 ± 23.41 ^a^	33.11 ± 10.85 ^a^	0.475
A22	3-methylbutyl octanoate	20.59	5.26 ± 2.78 ^a^	4.76 ± 2.82 ^a^	5.75 ± 3.49 ^a^	6.45 ± 3.04 ^a^	5.74 ± 0.97 ^a^	0.851
A23	Unknown terpene alcohol	21.18	1.80 ± 0.21 ^a^	1.51 ± 0.84 ^a^	1.73 ± 0.82 ^a^	2.02 ± 0.77 ^a^	1.96 ± 0.24 ^a^	0.475
A24	Ethyl 9-decenoate	21.23	0.50 ± 0.20 ^ab^	0.38 ± 0.46 ^b^	1.13 ± 0.74 ^ab^	1.24 ± 0.69 ^a^	0.81 ± 0.25 ^ab^	**0.025**
A25	Citronellol	22.76	4.43 ± 0.54 ^a^	3.76 ± 1.43 ^a^	4.18 ± 1.16 ^a^	4.34 ± 0.82 ^a^	4.86 ± 0.72 ^a^	0.195
A26	2,5-dimethyl-benzaldehyde	23.51	1.05 ± 0.15 ^a^	0.93 ± 0.33 ^a^	1.27 ± 0.79 ^a^	1.13 ± 0.81 ^a^	0.94 ± 0.13 ^a^	0.616
A27	Phenethyl acetate	23.65	11.25 ± 1.61 ^a^	10.30 ± 4.22 ^a^	9.90 ± 3.55 ^a^	10.68 ± 2.56 ^a^	12.43 ± 1.20 ^a^	0.326
A28	Hexanoic acid	24.26	6.53 ± 1.62 ^a^	6.28 ± 3.04 ^a^	5.79 ± 2.41 ^a^	6.48 ± 2.17 ^a^	6.41 ± 0.86 ^a^	0.931
A29	Geraniol	24.42	4.65 ± 0.70 ^a^	4.08 ± 2.16 ^a^	4.46 ± 1.98 ^a^	4.57 ± 1.42 ^a^	5.80 ± 0.77 ^a^	0.234
A30	Ethyl dodecanoate	24.57	3.66 ± 1.83 ^a^	5.31 ± 2.56 ^a^	6.29 ± 3.22 ^a^	7.62 ± 3.78 ^a^	5.32 ± 2.49 ^a^	0.238
A31	2,2,4-trimethyl-1,3-pentanediol isobutyrate	25.12	6.36 ± 1.79 ^a^	3.38 ± 1.74 ^a^	7.98 ± 10.28 ^a^	5.34 ± 2.78 ^a^	4.00 ± 2.00 ^a^	0.506
A32	Phenylethyl alcohol	25.57	132.81 ± 26.08 ^ab^	111.86 ± 36.43 ^b^	109.21 ± 36.29 ^b^	114.91 ± 29.59 ^b^	165.52 ± 37.94 ^a^	**0.015**
A33	Octanoic acid	28.52	61.51 ± 11.90 ^a^	49.54 ± 23.96 ^a^	52.63 ± 22.05 ^a^	57.76 ± 18.91 ^a^	53.54 ± 4.73 ^a^	0.605
**72 h**								
**Codes**	**Volatile organic compounds**	**RT**	**HF_HI**	**HF_LI**	**LF_HI**	**LF_LI**	**S**	***p* value**
A1	Ethyl acetate	2.43	24.44 ± 2.85 ^a^	21.92 ± 6.78 ^a^	22.71 ± 3.94 ^a^	21.79 ± 5.74 ^a^	22.73 ± 3.16 ^a^	0.707
A2	1,1-diethoxyethane	2.5	0.92 ± 0.22 ^a^	0.50 ± 0.18 ^a^	0.51 ± 0.24 ^a^	0.59 ± 0.15 ^a^	0.76 ± 0.29 ^a^	0.064
A3	Ethanol	3.08	740.27 ± 247.94 ^a^	1310.97 ± 209.72 ^a^	1310.10 ± 463.47 ^a^	1300.82 ± 372.64 ^a^	1135.00 ± 240.16 ^a^	0.099
A4	2,6-dimethyl-2-trans-6-octadiene	6	0.36 ± 0.07 ^a^	0.31 ± 0.11 ^a^	0.39 ± 0.07 ^a^	0.37 ± 0.13 ^a^	0.37 ± 0.07 ^a^	0.381
A5	2-methyl-1-propanol	6.12	19.80 ± 2.75 ^a^	15.15 ± 5.50 ^b^	17.65 ± 4.23 ^ab^	15.12 ± 5.04 ^b^	19.58 ± 3.74 ^a^	**0.006**
A6	Isoamyl acetate	6.76	58.05 ± 8.22 ^a^	56.42 ± 17.24 ^a^	56.68 ± 10.06 ^a^	58.03 ± 15.25 ^a^	53.84 ± 5.66 ^a^	0.898
A7	2-methylbutyl isobutyrate	8.68	0.22 ± 0.27 ^a^	0.56 ± 0.87 ^a^	0.43 ± 0.50 ^a^	0.81 ± 0.72 ^a^	0.30 ± 0.32 ^a^	0.211
A8	2-methyl-1-butanol	9.18	93.53 ± 10.61 ^a^	75.63 ± 26.06 ^a^	91.52 ± 17.30 ^a^	76.66 ± 22.75 ^a^	94.33 ± 13.22 ^a^	0.109
A9	3-methyl-1-butanol	9.23	167.47 ± 18.81 ^a^	138.82 ± 47.60 ^ab^	168.48 ± 30.92 ^a^	143.39 ± 42.43 ^ab^	169.53 ± 23.21 ^a^	**0.044**
A10	Ethyl hexanoate	9.84	33.64 ± 7.39 ^a^	26.96 ± 11.22 ^a^	32.22 ± 5.39 ^a^	32.28 ± 12.08 ^a^	30.99 ± 4.55 ^a^	0.417
A11	Methyl 4-methylenehexanoate	12.42	0.78 ± 0.30 ^a^	0.56 ± 0.39 ^a^	0.66 ± 0.17 ^a^	0.68 ± 0.33 ^a^	0.62 ± 0.19 ^a^	0.29
A12	Ethyl heptanoate	12.53	1.48 ± 0.34 ^a^	0.79 ± 0.52 ^b^	1.02 ± 0.28 ^ab^	0.95 ± 0.65 ^ab^	1.34 ± 0.35 ^ab^	**0.008**
A13	1-hexanol	12.95	0.58 ± 0.10 ^ab^	0.42 ± 0.21 ^b^	0.58 ± 0.15 ^a^	0.46 ± 0.20 ^ab^	0.56 ± 0.12 ^ab^	**0.013**
A14	Ethyl octanoate	15.34	146.18 ± 30.36 ^a^	113.89 ± 52.05 ^a^	114.68 ± 21.34 ^a^	117.91 ± 58.06 ^a^	121.55 ± 18.09 ^a^	0.375
A15	1-heptanol	15.85	1.68 ± 0.71 ^ab^	0.86 ± 0.39 ^b^	1.28 ± 0.52 ^ab^	1.17 ± 0.41 ^ab^	2.29 ± 1.29 ^a^	**0.03**
A16	Ethyl-1-hexanol	16.99	0.82 ± 0.11 ^a^	0.82 ± 0.20 ^a^	0.68 ± 0.13 ^a^	1.13 ± 1.38 ^a^	0.62 ± 0.13 ^a^	0.629
A17	1,2-dihydrolinalool	17.55	7.04 ± 1.17 ^a^	4.87 ± 1.96 ^b^	6.16 ± 1.81 ^ab^	5.52 ± 1.96 ^ab^	6.23 ± 0.72 ^ab^	**0.017**
A18	Linalool	17.85	36.63 ± 2.51 ^a^	26.68 ± 10.12 ^a^	34.07 ± 3.46 ^a^	31.87 ± 9.26 ^a^	33.77 ± 3.06 ^a^	0.126
A19	1-octanol	18.1	4.04 ± 0.50 ^a^	2.98 ± 1.09 ^b^	3.49 ± 0.49 ^ab^	3.25 ± 1.03 ^ab^	3.55 ± 0.36 ^ab^	**0.05**
A20	2-methylpropanoic acid	18.16	1.82 ± 0.20 ^a^	1.41 ± 0.63 ^a^	1.72 ± 0.29 ^a^	1.52 ± 0.50 ^a^	1.80 ± 0.25 ^a^	0.099
A21	Ethyl decanoate	20.14	28.83 ± 4.75 ^a^	25.07 ± 12.31 ^a^	24.06 ± 2.88 ^a^	21.54 ± 10.56 ^a^	23.90 ± 2.69 ^a^	0.549
A22	3-methylbutyl octanoate	20.59	8.43 ± 1.93 ^a^	6.04 ± 3.04 ^a^	6.41 ± 1.80 ^a^	6.16 ± 3.34 ^a^	6.71 ± 0.96 ^a^	0.269
A23	Unknown terpene alcohol	21.18	1.88 ± 0.16 ^a^	1.28 ± 0.79 ^a^	1.69 ± 0.19 ^a^	1.48 ± 0.71 ^a^	1.65±0.29 ^a^	0.269
A24	Ethyl 9-decenoate	21.23	1.31 ± 0.33 ^a^	0.81 ± 0.42 ^a^	0.63 ± 0.28 ^a^	0.74 ± 0.51 ^a^	0.98 ± 0.55 ^a^	0.064
A25	Citronellol	22.76	6.14 ± 0.70 ^a^	4.36 ± 1.64 ^b^	5.62 ± 0.62 ^ab^	5.04 ± 1.48 ^ab^	5.92 ± 0.97 ^ab^	**0.022**
A26	3,5-dimethyl-benzaldehyde	23.51	1.29 ± 0.14 ^a^	1.30 ± 0.19 ^a^	1.21 ± 0.21 ^a^	1.18 ± 0.20 ^a^	1.18 ± 0.16 ^a^	0.628
A27	Phenethyl acetate	23.65	16.43 ± 1.22 ^a^	12.71 ± 5.70 ^a^	15.20 ± 1.33 ^a^	14.44 ± 4.80 ^a^	16.26 ± 1.32 ^a^	0.275
A28	Hexanoic acid	24.26	7.18 ± 0.76 ^a^	5.79 ± 2.99 ^a^	7.29 ± 1.44 ^a^	6.85 ± 2.92 ^a^	6.82 ± 0.59 ^a^	0.612
A29	Geraniol	24.42	5.57 ± 0.41 ^a^	3.86 ± 2.05 ^a^	5.47 ± 0.82 ^a^	4.55 ± 2.08 ^a^	5.54 ± 0.58 ^a^	0.128
A30	Ethyl dodecanoate	24.57	1.64 ± 0.51 ^ab^	2.80 ± 1.24 ^a^	2.30 ± 0.27 ^ab^	1.84 ± 0.91 ^ab^	1.42 ± 0.43 ^b^	**0.047**
A31	2,2,4-trimethyl-1,3-pentanediol isobutyrate	25.12	2.44 ± 1.09 ^a^	4.84 ± 3.19 ^a^	3.17 ± 1.70 ^a^	4.85 ± 6.10 ^a^	1.48 ± 0.61 ^a^	0.333
A32	Phenylethyl alcohol	25.57	182.84 ± 28.54 ^ab^	122.76 ± 47.69 ^c^	168.93 ± 24.59 ^ab^	145.12 ± 45.33 ^bc^	192.80 ± 39.75 ^a^	**0.001**
A33	Octanoic acid	28.52	49.92 ± 5.05 ^a^	41.98 ± 22.27 ^a^	54.63 ± 9.25 ^a^	50.18 ± 21.84 ^a^	49.70 ± 3.98 ^a^	0.601
**96 h**								
**Codes**	**Volatile organic compounds**	**RT**	**HF_HI**	**HF_LI**	**LF_HI**	**LF_LI**	**S**	***p* value**
A1	Ethyl acetate	2.43	27.35 ± 5.45 ^a^	27.18 ± 6.37 ^a^	25.99 ± 7.45 ^a^	24.47 ± 4.75 ^a^	23.16 ± 2.76 ^a^	0.299
A2	1,1-diethoxyethane	2.5	1.36 ± 0.60 ^a^	1.20 ± 0.39 ^a^	1.26 ± 0.20 ^a^	1.26 ± 0.64 ^a^	1.21 ± 0.36 ^a^	0.987
A3	Ethanol	3.08	1287.99 ± 181.22 ^a^	1232.84 ± 284.62 ^a^	1175.28 ± 134.38 ^a^	1427.62 ± 193.39 ^a^	1130.10 ± 84.85 ^a^	0.083
A4	2,6-dimethyl-2-trans-6-octadiene	6	0.35 ± 0.04 ^a^	0.33 ± 0.07 ^a^	0.35 ± 0.03 ^a^	0.35 ± 0.06 ^a^	0.32 ± 0.05 ^a^	0.59
A5	2-methyl-1-propanol	6.12	19.61 ± 3.97 ^a^	18.42 ± 4.44 ^a^	20.02 ± 2.65 ^a^	17.07 ± 3.28 ^a^	19.23 ± 4.14 ^a^	0.084
A6	Isoamyl acetate	6.76	59.13 ± 9.30 ^a^	61.80 ± 14.66 ^a^	54.61 ± 23.24 ^a^	58.80 ± 10.08 ^a^	47.60 ± 8.89 ^a^	0.253
A7	2-methylbutyl isobutyrate	8.68	0.23 ± 0.27 ^a^	0.33 ± 0.25 ^a^	0.18 ± 0.30 ^a^	0.70 ± 0.56 ^a^	0.31 ± 0.32 ^a^	0.103
A8	2-methyl-1-butanol	9.18	95.18 ± 13.65 ^a^	91.15 ± 16.82 ^a^	95.18 ± 10.22 ^a^	85.66 ± 11.81 ^a^	91.81 ± 14.40 ^a^	0.052
A9	3-methyl-1-butanol	9.23	169.12 ± 24.70 ^a^	164.66 ± 30.28 ^a^	171.66 ± 18.47 ^a^	157.72 ± 19.67 ^a^	163.56 ± 25.10 ^a^	0.16
A10	Ethyl hexanoate	9.84	35.72 ± 11.35 ^a^	33.42 ± 6.90 ^a^	30.61 ± 13.81 ^a^	35.89 ± 6.08 ^a^	28.28 ± 5.05 ^a^	0.29
A11	Methyl 4-methylenehexanoate	12.42	0.65 ± 0.20 ^ab^	0.55 ± 0.19 ^ab^	0.55 ± 0.27 ^ab^	0.66 ± 0.18 ^a^	0.45 ± 0.16 ^b^	**0.039**
A12	Ethyl heptanoate	12.53	1.63 ± 0.46 ^a^	1.29 ± 0.27 ^a^	1.24 ± 0.52 ^a^	1.34 ± 0.35 ^a^	1.37 ± 0.36 ^a^	0.329
A13	1-hexanol	12.95	0.65 ± 0.10 ^a^	0.59 ± 0.13 ^ab^	0.63 ± 0.12 ^ab^	0.54 ± 0.09 ^b^	0.59 ± 0.13 ^ab^	**0.019**
A14	Ethyl octanoate	15.34	150.21 ± 42.30 ^a^	125.66 ± 20.08 ^a^	135.81 ± 51.52 ^a^	140.33 ± 22.87 ^a^	118.90 ± 14.46 ^a^	0.145
A15	1-heptanol	15.85	1.78 ± 0.65 ^a^	1.72 ± 0.29 ^a^	2.15 ± 0.85 ^a^	2.22 ± 0.72 ^a^	2.72 ± 1.42 ^a^	0.482
A16	Ethyl-1-hexanol	16.99	0.63 ± 0.21 ^a^	0.54 ± 0.19 ^a^	0.59 ± 0.06 ^a^	0.54 ± 0.08 ^a^	0.60 ± 0.12 ^a^	0.597
A17	1,2-dihydrolinalool	17.55	6.95 ± 0.48 ^a^	6.18 ± 1.08 ^a^	7.15 ± 0.55 ^a^	6.14 ± 1.24 ^a^	6.12 ± 0.81 ^a^	0.159
A18	Linalool	17.85	34.92 ± 2.29 ^a^	30.26 ± 3.02 ^a^	34.16 ± 3.19 ^a^	34.07 ± 3.37 ^a^	30.52 ± 3.56 ^a^	0.14
A19	1-octanol	18.1	4.10 ± 0.52 ^a^	3.48 ± 0.39 ^b^	3.91 ± 0.61 ^ab^	3.73 ± 0.34 ^ab^	3.52 ± 0.33 ^b^	**0.002**
A20	2-methylpropanoic acid	18.16	1.79 ± 0.33 ^a^	1.67 ± 0.29 ^a^	1.78 ± 0.20 ^a^	1.75 ± 0.17 ^a^	1.78 ± 0.23 ^a^	0.548
A21	Ethyl decanoate	20.14	25.38 ± 3.70 ^a^	22.64 ± 6.17 ^a^	30.36 ± 9.95 ^a^	25.92 ± 3.55 ^a^	25.42 ± 6.36 ^a^	0.256
A22	3-methylbutyl octanoate	20.59	8.57 ± 2.38 ^a^	6.99± 0.71 ^a^	7.77 ± 2.45 ^a^	7.88 ± 1.62 ^a^	6.80 ± 1.05 ^a^	0.128
A23	Unknown terpene alcohol	21.18	1.51 ± 0.16 ^a^	1.46 ± 0.15 ^a^	1.68 ± 0.18 ^a^	1.74 ± 0.21 ^a^	1.63 ± 0.26 ^a^	0.181
A24	Ethyl 9-decenoate	21.23	1.71 ± 0.96 ^a^	1.12 ± 0.25 ^a^	1.41 ± 0.85 ^a^	1.79 ± 1.02 ^a^	2.19 ± 1.32 ^a^	0.565
A25	Citronellol	22.76	6.21 ± 0.56 ^a^	5.51 ± 0.54 ^a^	6.37 ± 0.30 ^a^	6.21 ± 0.82 ^a^	6.19 ± 1.16 ^a^	0.49
A26	3,5-dimethyl-benzaldehyde	23.51	1.59 ± 0.50 ^a^	1.22 ± 0.20 ^ab^	1.24 ± 0.17 ^ab^	1.19 ± 0.13 ^b^	1.20 ± 0.13 ^ab^	**0.035**
A27	Phenethyl acetate	23.65	16.21 ± 1.78 ^a^	15.77 ± 1.69 ^a^	16.90 ± 0.96 ^a^	16.47 ± 0.73 ^a^	15.52 ±1.40 ^a^	0.274
A28	Hexanoic acid	24.26	7.59 ± 1.30 ^ab^	7.45 ± 1.33 ^ab^	8.17 ± 0.84 ^a^	7.90 ± 0.81 ^ab^	7.02 ± 0.91 ^b^	**0.054**
A29	Geraniol	24.42	4.80 ± 0.40 ^a^	4.25 ± 0.45 ^a^	4.95 ± 0.49 ^a^	4.75 ± 0.63 ^a^	4.61 ± 0.75 ^a^	0.204
A30	Ethyl dodecanoate	24.57	0.95 ± 0.32 ^a^	1.24 ± 0.35 ^a^	0.97 ± 0.36 ^a^	1.24 ± 0.20 ^a^	1.13 ± 0.23 ^a^	0.408
A31	2,2,4-trimethyl-1,3-pentanediol isobutyrate	25.12	2.48 ± 1.00 ^a^	3.28 ± 0.99 ^a^	1.71 ± 1.64 ^a^	1.40 ± 0.60 ^a^	1.89 ± 0.83 ^a^	0.071
A32	Phenylethyl alcohol	25.57	171.23 ± 18.80 ^a^	159.30 ± 29.03 ^a^	169.27 ± 14.90 ^a^	166.47 ± 24.08 ^a^	195.27 ± 44.58 ^a^	0.229
A33	Octanoic acid	28.52	50.40 ± 4.93 ^b^	51.39 ± 8.83 ^ab^	57.19 ± 6.04 ^a^	54.35 ± 4.88 ^ab^	51.83 ± 8.47 ^ab^	**0.024**
**120 h**								
**Codes**	**Volatile organic compounds**	**RT**	**HF_HI**	**HF_LI**	**LF_HI**	**LF_LI**	**S**	***p* value**
A1	Ethyl acetate	2.43	25.37 ± 5.43 ^a^	25.89 ± 3.41 ^a^	25.07 ± 4.65 ^a^	26.23 ± 5.15 ^a^	26.41 ± 5.08 ^a^	0.976
A2	1,1-diethoxyethane	2.5	1.92 ± 1.14 ^a^	2.01 ± 0.98 ^a^	2.58 ± 1.27 ^a^	1.35 ± 0.84 ^a^	3.05 ± 1.36 ^a^	0.226
A3	Ethanol	3.08	1191.93 ± 178.7 ^a^	1289.36 ± 183.16 ^a^	1342.30 ± 191.47 ^a^	1185.40 ± 282.46 ^a^	665.06 ± 251.01 ^b^	**0.001**
A4	2,6-dimethyl-2-trans-6-octadiene	6	0.31 ± 0.06 ^a^	0.30 ± 0.03 ^a^	0.30 ± 0.04 ^a^	0.34 ± 0.07 ^a^	0.32 ± 0.05 ^a^	0.44
A5	2-methyl-1-propanol	6.12	17.92 ± 3.33 ^a^	18.75 ± 2.51 ^a^	18.21 ± 3.91 ^a^	16.87 ± 2.30 ^a^	21.51 ± 5.08 ^a^	0.181
A6	Isoamyl acetate	6.76	53.63 ± 11.56 ^a^	54.81 ± 8.44 ^a^	52.12 ± 8.36 ^a^	56.01 ± 10.71 ^a^	49.07 ± 6.81 ^a^	0.499
A7	2-methylbutyl isobutyrate	8.68	0.41 ± 0.35 ^a^	0.35 ± 0.58 ^a^	0.22 ± 0.16 ^a^	0.29 ± 0.32 ^a^	0.14 ± 0.09 ^a^	0.637
A8	2-methyl-1-butanol	9.18	89.24 ± 11.73 ^a^	89.29 ± 7.89 ^a^	91.38 ± 13.11 ^a^	84.54 ± 9.30 ^a^	97.99 ± 14.60 ^a^	0.313
A9	3-methyl-1-butanol	9.23	159.86 ± 22.09 ^a^	159.90 ± 13.89 ^a^	161.60 ± 22.94 ^a^	152.38 ± 15.88 ^a^	172.08 ± 25.11 ^a^	0.446
A10	Ethyl hexanoate	9.84	33.04 ± 9.26 ^a^	30.88 ± 7.32 ^a^	33.92 ± 6.72 ^a^	30.06 ± 5.41 ^a^	31.96 ± 7.07 ^a^	0.684
A11	Methyl 4-methylenehexanoate	12.42	0.55 ± 0.19 ^a^	0.49 ± 0.19 ^a^	0.43 ± 0.14 ^a^	0.50 ± 0.12 ^a^	0.46 ± 0.16 ^a^	0.295
A12	Ethyl heptanoate	12.53	1.50 ± 0.41 ^a^	1.30 ± 0.57 ^a^	1.61 ± 0.45 ^a^	1.31 ± 0.27 ^a^	2.15 ± 0.70 ^a^	0.078
A13	1-hexanol	12.95	0.59 ± 0.12 ^a^	0.59 ± 0.06 ^a^	0.55 ± 0.11 ^a^	0.58 ± 0.09 ^a^	0.63 ± 0.10 ^a^	0.611
A14	Ethyl octanoate	15.34	140.38 ± 32.33 ^a^	138.81 ± 24.08 ^a^	139.58 ± 19.90 ^a^	124.72 ± 22.07 ^a^	151.92 ± 41.78 ^a^	0.385
A15	1-heptanol	15.85	2.00 ± 0.62 ^a^	2.25 ± 0.21 ^a^	2.44 ± 1.01 ^a^	2.31 ± 0.69 ^a^	3.61 ± 1.34 ^a^	0.08
A16	Ethyl-1-hexanol	16.99	0.60 ± 0.09 ^ab^	0.60 ± 0.20 ^ab^	0.50 ± 0.12 ^b^	0.50 ± 0.08 ^b^	0.75 ± 0.09 ^a^	**0.026**
A17	1,2-dihydrolinalool	17.55	6.67 ± 1.11 ^a^	5.73 ± 0.79 ^a^	6.57 ± 1.88 ^a^	6.46 ± 1.19 ^a^	7.25 ± 1.62 ^a^	0.506
A18	Linalool	17.85	31.38 ± 2.61 ^a^	30.39 ± 1.35 ^a^	29.42 ± 1.34 ^a^	28.93 ± 4.13 ^a^	32.28 ± 3.68 ^a^	0.302
A19	1-octanol	18.1	3.90 ± 0.47 ^a^	3.60 ± 0.50 ^a^	3.66 ± 0.34 ^a^	3.47 ± 0.49 ^a^	4.05 ± 0.69 ^a^	0.225
A20	2-methylpropanoic acid	18.16	1.71 ± 0.31 ^ab^	1.73 ± 0.16 ^ab^	1.68 ± 0.24 ^ab^	1.54 ± 0.13 ^b^	1.99 ± 0.33 ^a^	**0.04**
A21	Ethyl decanoate	20.14	29.29 ± 5.97 ^a^	31.18 ± 10.35 ^a^	33.26 ± 11.81 ^a^	28.10 ± 3.65 ^a^	39.54 ± 14.14 ^a^	0.402
A22	3-methylbutyl octanoate	20.59	8.43 ± 2.04 ^a^	8.34 ± 1.80 ^a^	8.58 ± 1.33 ^a^	7.09 ± 1.65 ^a^	9.91 ± 3.13 ^a^	0.203
A23	Unknown terpene alcohol	21.18	1.53 ± 0.18 ^a^	1.58 ± 0.11 ^a^	1.45 ± 0.06 ^a^	1.35 ± 0.26 ^a^	1.60 ± 0.24 ^a^	0.211
A24	Ethyl 9-decenoate	21.23	3.07 ± 1.56 ^a^	3.27 ± 0.69 ^a^	3.20 ± 1.59 ^a^	2.32 ± 1.23 ^a^	4.85 ± 2.39 ^a^	0.217
A25	Citronellol	22.76	6.72 ± 0.66 ^a^	6.40 ± 0.34 ^a^	6.28 ± 0.60 ^a^	6.08 ± 0.76 ^a^	7.41 ± 1.25 ^a^	0.066
A26	3,5-dimethyl-benzaldehyde	23.51	1.47 ± 0.20 ^a^	1.42 ± 0.22 ^a^	1.38 ± 0.27 ^a^	1.20 ± 0.27 ^a^	1.49 ± 0.27 ^a^	0.093
A27	Phenethyl acetate	23.65	15.31 ± 1.80 ^a^	15.28 ± 0.84 ^a^	14.46 ± 1.35 ^a^	13.87 ± 2.07 ^a^	15.57 ± 1.51 ^a^	0.313
A28	Hexanoic acid	24.26	7.83 ± 1.37 ^a^	7.94 ± 1.09 ^a^	7.63 ± 1.38 ^a^	7.34 ± 1.58 ^a^	7.49 ± 0.69 ^a^	0.859
A29	Geraniol	24.42	4.28 ± 0.34 ^a^	4.20 ± 0.23 ^a^	3.72 ± 0.54 ^a^	3.81 ± 0.96 ^a^	4.34 ± 0.47 ^a^	0.185
A30	Ethyl dodecanoate	24.57	0.72 ± 0.18 ^c^	1.08 ± 0.24 ^ab^	0.76 ± 0.06 ^c^	1.32 ± 0.20 ^a^	0.86 ± 0.19 ^bc^	**0.001**
A31	2,2,4-trimethyl-1,3-pentanediol isobutyrate	25.12	3.57 ± 1.50 ^a^	3.84 ± 2.62 ^a^	1.87 ± 1.34 ^a^	4.62 ± 2.24 ^a^	4.21 ± 1.59 ^a^	0.212
A32	Phenylethyl alcohol	25.57	181.37 ± 23.04 ^ab^	173.29 ± 18.69 ^ab^	167.33 ± 21.83 ^ab^	148.65 ± 23.78 ^b^	211.75 ± 38.30 ^a^	**0.021**
A33	Octanoic acid	28.52	57.01 ± 5.19 ^a^	58.15 ± 12.53 ^a^	54.35 ± 8.74 ^a^	56.70 ± 8.33 ^a^	50.18 ± 5.69 ^a^	0.388
**144 h**								
**Codes**	**Volatile organic compounds**	**RT**	**HF_HI**	**HF_LI**	**LF_HI**	**LF_LI**	**S**	***p* values**
A1	Ethyl acetate	2.43	25.91 ± 3.55 ^a^	29.44 ± 5.98 ^a^	26.16 ± 4.57 ^a^	28.07 ± 3.04 ^a^	25.83 ± 3.61 ^a^	0.062
A2	1,1-diethoxyethane	2.5	4.05 ± 2.24 ^a^	4.43 ± 1.18 ^a^	3.44 ± 0.82 ^a^	3.43 ± 2.06 ^a^	4.39 ± 0.94 ^a^	0.714
A3	Ethanol	3.08	1306.27 ± 384.06 ^a^	1305.65 ± 295.92 ^a^	1037.67 ± 270.81 ^a^	941.88 ± 304.17 ^a^	1300.48 ± 261.20 ^a^	0.11
A4	2,6-dimethyl-2-trans-6-octadiene	6	0.33 ± 0.05 ^a^	0.30 ± 0.05 ^a^	0.28 ± 0.03 ^a^	0.31 ± 0.04 ^a^	0.33 ± 0.08 ^a^	0.302
A5	2-methyl-1-propanol	6.12	18.62 ± 3.50 ^a^	19.86 ± 4.11 ^a^	18.39 ± 3.66 ^a^	19.38 ± 2.64 ^a^	20.74 ± 4.06 ^a^	0.604
A6	Isoamyl acetate	6.76	51.17 ± 8.68 ^a^	57.09 ± 11.37 ^a^	52.07 ± 7.45 ^a^	55.88 ± 8.26 ^a^	48.42 ± 8.30 ^a^	0.083
A7	2-methylbutyl isobutyrate	8.68	0.30 ± 0.33 ^a^	0.16 ± 0.14 ^a^	0.19 ± 0.29 ^a^	0.24 ± 0.30 ^a^	0.24 ± 0.31 ^a^	0.888
A8	2-methyl-1-butanol	9.18	90.63 ± 12.19 ^a^	94.94 ± 15.00 ^a^	90.22 ± 13.66 ^a^	92.78 ± 10.19 ^a^	94.93 ± 12.49 ^a^	0.816
A9	3-methyl-1-butanol	9.23	161.54 ± 21.74 ^a^	168.26 ± 25.95 ^a^	161.47 ± 23.41 ^a^	166.86 ± 20.09 ^a^	167.97 ± 22.07 ^a^	0.801
A10	Ethyl hexanoate	9.84	33.54 ± 8.04 ^abc^	35.90 ± 8.77 ^ab^	30.02 ± 6.24 ^bc^	38.05 ± 9.47 ^a^	28.20 ± 5.75 ^c^	**0.001**
A11	Methyl 4-methylenehexanoate	12.42	0.51 ± 0.17 ^a^	0.55 ± 0.24 ^a^	0.44 ± 0.12 ^a^	0.56 ± 0.15 ^a^	0.47 ± 0.21 ^a^	0.173
A12	Ethyl heptanoate	12.53	1.70 ± 0.35 ^a^	1.77 ± 0.52 ^a^	1.40 ± 0.33 ^a^	1.98 ± 0.29 ^a^	1.83 ± 0.45 ^a^	0.106
A13	1-hexanol	12.95	0.60 ± 0.10 ^a^	0.63 ± 0.14 ^a^	0.62 ± 0.12 ^a^	0.61 ± 0.10 ^a^	0.63 ± 0.09 ^a^	0.912
A14	Ethyl octanoate	15.34	147.42 ± 25.51 ^abc^	154.11 ± 44.10 ^ab^	122.17 ± 15.91 ^c^	161.12 ± 27.16 ^a^	129.03 ± 22.47 ^bc^	**0.004**
A15	1-heptanol	15.85	2.14 ± 0.70 ^a^	2.49 ± 0.36 ^a^	2.54 ± 0.93 ^a^	2.74 ± 0.70 ^a^	3.75 ± 1.33 ^a^	0.093
A16	Ethyl-1-hexanol	16.99	0.65 ± 0.10 ^a^	0.70 ± 0.15 ^a^	0.68 ± 0.11 ^a^	0.62 ± 0.16 ^a^	0.70 ± 0.07 ^a^	0.626
A17	1,2-dihydrolinalool	17.55	6.48 ± 0.72 ^a^	6.65 ± 1.00 ^a^	6.40 ± 0.66 ^a^	6.81 ± 0.39 ^a^	6.80 ± 0.70 ^a^	0.765
A18	Linalool	17.85	31.91 ± 2.22 ^a^	32.78± 4.15 ^a^	30.28 ± 2.08 ^a^	33.16 ± 1.76 ^a^	34.04 ± 3.31 ^a^	0.219
A19	1-octanol	18.1	3.99 ± 0.22 ^ab^	3.96 ± 0.58 ^ab^	3.54 ± 0.30 ^b^	4.03 ± 0.33 ^a^	3.76 ± 0.36 ^ab^	**0.036**
A20	2-methylpropanoic acid	18.16	1.72 ± 0.20 ^a^	1.84 ± 0.29 ^a^	1.65 ± 0.19 ^a^	1.81 ± 0.27 ^a^	1.88 ± 0.31 ^a^	0.188
A21	Ethyl decanoate	20.14	27.49 ± 6.43 ^a^	28.11 ± 5.31 ^a^	26.12 ± 6.61 ^a^	29.91 ± 4.28 ^a^	31.56 ± 10.96 ^a^	0.764
A22	3-methylbutyl octanoate	20.59	9.03 ± 1.45 ^ab^	9.13 ± 2.27 ^ab^	7.23 ± 0.73 ^b^	9.89 ± 1.97 ^a^	7.80 ± 1.26 ^b^	**0.006**
A23	Unknown terpene alcohol	21.18	1.45 ± 0.17 ^a^	1.52 ± 0.13 ^a^	1.36 ± 0.07 ^a^	1.48 ± 0.10 ^a^	1.54 ± 0.15 ^a^	0.212
A24	Ethyl 9-decenoate	21.23	4.19 ± 2.12 ^a^	4.51 ± 0.48 ^a^	3.67 ± 1.22 ^a^	4.65 ± 1.22 ^a^	5.63 ± 2.34 ^a^	0.438
A25	Citronellol	22.76	7.12 ± 0.87 ^a^	7.25 ± 0.72 ^a^	6.87 ± 0.46 ^a^	7.03 ± 0.57 ^a^	8.15 ± 1.13 ^a^	0.072
A26	3,5-dimethyl-benzaldehyde	23.51	1.54 ± 0.15 ^b^	1.60 ± 0.15 ^b^	1.48 ± 0.32 ^b^	1.58 ± 0.19 ^b^	1.94 ± 0.29 ^a^	**0.004**
A27	Phenethyl acetate	23.65	14.44 ± 0.60 ^ab^	15.84 ± 1.11 ^a^	13.98 ± 0.71 ^b^	14.86 ± 1.25 ^ab^	15.57 ± 1.27 ^ab^	**0.030**
A28	Hexanoic acid	24.26	7.63 ± 0.83 ^a^	8.45 ± 1.14 ^a^	7.64 ± 0.79 ^a^	8.21 ± 1.44 ^a^	7.73 ± 0.92 ^a^	0.313
A29	Geraniol	24.42	3.99 ± 0.50 ^a^	4.07 ± 0.41 ^a^	3.93 ± 0.44 ^a^	3.97 ± 0.35 ^a^	4.42 ± 0.34 ^a^	0.231
A30	Ethyl dodecanoate	24.57	0.64 ± 0.09 ^b^	0.65 ± 0.16 ^b^	0.69 ± 0.10 ^ab^	0.93 ± 0.28 ^a^	0.53 ± 0.06 ^b^	**0.002**
A31	2,2,4-trimethyl-1,3-pentanediol isobutyrate	25.12	2.41 ±1.11 ^a^	2.29 ± 0.89 ^a^	2.52 ± 1.08 ^a^	3.15 ± 0.77 ^a^	3.19 ± 1.31 ^a^	0.476
A32	Phenylethyl alcohol	25.57	177.95 ± 24.31 ^ab^	180.54 ± 23.76 ^ab^	168.47 ± 15.58 ^b^	162.71 ± 21.40 ^b^	215.52 ± 33.77 ^a^	**0.015**
A33	Octanoic acid	28.52	55.70 ± 11.85 ^a^	54.33 ± 8.39 ^a^	53.21 ± 7.94 ^a^	52.42 ± 9.40 ^a^	51.41 ± 5.37 ^a^	0.840

Values presented are means ± standard deviations of 6 measurements (3 fermentation replicates × 2 analytical replicates). Different letters in the same row indicate statistically significant differences (*p* < 0.05) by Tukey posthoc multiple comparison test. Not detected (ND). Bolded *p* values are significantly different.
