# Supplementary material for: The Effect of Sound Frequency and Intensity on Yeast Growth, Fermentation Performance and Volatile Composition of Beer
Source: Molecules. 2021 Nov 29;26(23):7239. doi: 10.3390/molecules26237239 (PMC8658799; doi:10.3390/molecules26237239)
Supplement: Supplementary file 1 [file molecules-26-07239-s001.zip › Adadi et al., 2021_supplementaryinfo_26112021.pdf]

molecules

MDPI

Supplementary material

The Effect of Sound Frequency and Intensity on Yeast Growth, Fermentation Performance and Volatile Composition of Beer

Parise Adadi [1], Alastair Harris [2], Phil Bremer [1], Patrick Silcock [1], Austen R.D. Ganley [2], Andrew G. Jeffs [2,3] and Graham T. Eyres [1,]*

[1] Department of Food Science, University of Otago, P.O. Box 56, Dunedin 9054, New Zealand; parise.adadi@postgrad.otago.ac.nz (P.A.); phil.bremer@otago.ac.nz (P.B.); pat.silcock@otago.ac.nz (P.S.)
[2] School of Biological Sciences, University of Auckland, Auckland, 1142, New Zealand; ahar394@auck-landuni.ac.nz (A.H.); a.ganley@auckland.ac.nz (A.R.D.G.)
[3] Institute of Marine Science, University of Auckland, Private Bag, 92019 Auckland, New Zealand; a.jeffs@auckland.ac.nz (A.G.J.)

Table S1. Viable yeast numbers in suspension (× 10^6 cells/mL) during fermentation for five treatment conditions.

Sampling time (h)	LF_LI (× 10^6 cells/mL)	LF_HI (× 10^6 cells/mL)	HF_LI (× 10^6 cells/mL)	HF_HI (× 10^6 cells/mL)	S (× 10^6 cells/mL)
0	9.10 (0.22)$_c^A$	9.10 (0.22)$_c^A$	9.10 (0.22)$_c^A$	9.10 (0.22)$_c^A$	9.10 (0.22)$_d^A$
10	16.88 (2.27)$_b^A$	16.71 (2.48)$_b^A$	18.31 (4.09)$_b^A$	18.78 (2.15)$_b^A$	21.88 (2.49)$_b^B$
24	35.31 (1.00)$_a^B$	34.33 (4.11)$_a^B$	34.86 (2.90)$_a^B$	38.98 (3.64)$_a^{AB}$	44.05 (1.58)$_a^A$
48	13.22 (1.01)$_{bc}^A$	15.67 (3.12)$_{bc}^A$	12.05 (2.36)$_c^A$	12.76 (1.67)$_c^A$	14.47 (1.75)$_c^A$
72	11.50 (4.44)$_c^A$	9.60 (3.42)$_c^A$	8.43 (1.98)$_c^A$	9.26 (2.15)$_c^A$	8.33 (0.95)$_d^A$
96	1.71 (0.46)$_d^A$	2.02 (0.46)$_d^A$	2.07 (0.88)$_d^A$	1.74 (0.09)$_d^A$	1.81 (0.40)$_e^A$
120	1.50 (0.61)$_d^A$	1.17 (0.15)$_d^A$	1.09 (0.41)$_d^A$	0.95 (0.29)$_d^A$	1.14 (0.57)$_e^A$
144	0.48 (0.11)$_d^A$	0.57 (0.31)$_d^A$	0.38 (0.23)$_d^A$	0.67 (0.21)$_d^A$	0.79 (0.37)$_e^A$

Low-frequency and low intensity (LF_LI); low-frequency and high-intensity (LF_HI); high-frequency and low-intensity (HF_LI); high-frequency and high-intensity (HF_HI); silence (S). Data represented are the means (± SD) of three fermentation replicates. Means with different lower-case letters (subscripts) denote significant differences ($p < 0.05$) in the same column (differences during fermentation time for the same treatment). Different capital letters (superscripts) denote significant differences ($p < 0.05$) in the same row (differences between treatments at a given fermentation time).

Table S2. Wort gravity (°P) during fermentation over time for five treatment conditions.

Sampling time (h)	LF_LI	LF_HI	HF_LI	HF_HI	S
0	12.03 (0.06)$_a^A$	12.03 (0.06)$_a^A$	12.03 (0.06)$_a^A$	12.03 (0.06)$_a^A$	12.03 (0.06)$_a^A$
10	10.87 (0.06)$_b^A$	10.87 (0.06)$_b^A$	10.83 (0.06)$_b^A$	10.77 (0.06)$_b^A$	10.80 (0.10)$_b^A$
24	8.13 (0.06)$_c^A$	8.17 (0.12)$_c^A$	7.80 (0.10)$_c^B$	7.83 (0.06)$_c^B$	7.57 (0.15)$_c^B$
48	3.93 (0.32)$_d^{AB}$	4.10 (0.00)$_d^A$	3.73 (0.12)$_d^{AB}$	3.53 (0.25)$_d^{AB}$	3.20 (0.52)$_d^B$
72	2.57 (0.21)$_e^A$	2.57 (0.12)$_e^A$	2.63 (0.06)$_e^A$	2.47 (0.06)$_e^A$	2.50 (0.17)$_e^A$
96	2.30 (0.10)$_e^A$	2.23 (0.06)$_f^A$	2.33 (0.06)$_f^A$	2.30 (0.10)$_e^A$	2.30 (0.10)$_e^A$
120	2.20 (0.00)$_e^A$	2.20 (0.00)$_f^A$	2.20 (0.00)$_f^A$	2.23 (0.06)$_e^A$	2.20 (0.00)$_e^A$
144	2.20 (0.00)$_e^A$	2.20 (0.00)$_f^A$	2.20 (0.00)$_f^A$	2.17 (0.06)$_e^A$	2.17 (0.06)$_e^A$

Low-frequency and low intensity (LF_LI); low-frequency and high-intensity (LF_HI); high-frequency and low-intensity (HF_LI); high-frequency and high-intensity (HF_HI); silence (S). Data presented are the means (± SD) of three fermentation replicates. Means with different lower-case letters (subscripts) denote significant differences ($p < 0.05$) in the same column (differences during fermentation time for the same treatment). Different capital letters (superscripts) indicate significant differences ($p < 0.05$) in the same row (differences between treatments at a given fermentation time).

Supplementary Data S1. Bespoke MATLAB® scripts for sound generation.

```
Introduction='this script will produce noise within your specified frequency range, plot the response curves and save the audio to the current folder in matlab'; Introduction
fs = input('please insert your required sampling rate = ');
Fp1 = input('what is your lowest frequency required?');
Fp2 = input('what is your highest frequency required?');
Ast1 = input('what max power do you want your noise floor to be =');
Ast2 = Ast1;
Ap =  input('amount of ripple allowed in the pass band (set as 0.5 is unsure) =');
T = input('how long (seconds) do you want the audio to be?');
d1=fdesign.bandpass('Fst1,Fp1,Fp2,Fst2,Ast1,Ap,Ast2',Fp1-50,Fp1,Fp2,Fp2+50,Ast1,Ap,Ast2,fs);
Hd1 = design(d1);
y1 = filter(Hd1,randn(fs*T,1));
fvtool(Hd1);    %this will just plot your magnitude for you to look at.

audiowrite('test.wav',y1,fs);%this is going to save the noise as a .wav file, saved to the current folder in matlab.
                               %you can change the filename to whatever,
                               %but keep the ".wav" at the end.
```

Audio S1. Sound generated by Bespoke MATLAB® scripts (Supplementary Data 1) and saved as WAV. file (200-800 Hz; 800-2000 Hz (please see attached files "Audio files_Adadi et al., 2021.wav".

Supplementary Data S2. Bespoke MATLAB® scripts for calculating the mean level of sound intensity delivered before commencing fermentation. The blue arrow shows an uploaded recorded file 'RO7_0003.wav' for analysis. The red bolded numbers are the hydrophone calibrition numbers for input level 20 % (this varies according to individul hydrophone).

```
filename='RO7_0003.wav';
```

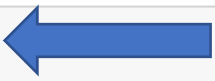

```
[dat,fs]=audioread(filename);
dat=dat(:,1);
n=1;

samr=fs;                    % sampling rate
nstart=n*samr;                        % starting point for plot/spectrum
nsam=fs;                          % number of samples

tlo=4; tup=15;                     % time window for plot  -  NB seconds

nlo=nstart+nsam*tlo+1;          % first data sample index
```

```
nup=nstart+nsam*tup;            % last data sample index

data=data(nlo:nup)';              % data segment for plot/spectrum
data=data*188516774.3;                      % apply calibration
npts=length(data);                   % number of points in data segment
tint=npts/samr;                      % time interval of data segment
t=tlo+(1:npts)/samr;            % time axis for plot

subplot(411);
plot(t,data);
%{
flo=20;         fhi=22000;

fdata=bpfilt(data,flo,fhi);

subplot(412);
plot(t,fdata);
%}

subplot(413);
pfdata=abs(data).^2;                                % is Prms^2 of filtered data
smp=smooth(pfdata,5);
dbpd=10*log10(smp);                                 % converts into dB
plot(t,dbpd);
dbav=10*log10(sum(pfdata)/length(pfdata));
%axis([min(t),max(t),min(dbpd),max(dbpd)]);

spec=fft(data)/npts;                         % fft of data segment NB Matlab fft drops 1/N
fnc=1/tint;                                 % frequency increment in spectrum
nfreq=floor(npts/2);                          % number of frequencies in spectrum
f=fnc*(0:nfreq-1)/1000;                     % frequencies for plot  -  /1000 for kHz
pspec=abs(spec(1:nfreq)).^2;                % power spectrum
pspec=pspec/fnc;
dbspec=10*log10(pspec);

subplot(414);                                            % spectrum of filtered data
smdbspec=10*log10(smooth(pspec,10));
semilogx(f,smdbspec);
axis([0.1,24,min(smdbspec),max(smdbspec)]);
%axis([0.1,24,-120,-20]);
title(['Mean level = ',num2str(dbav)]);
figure(gcf)
```

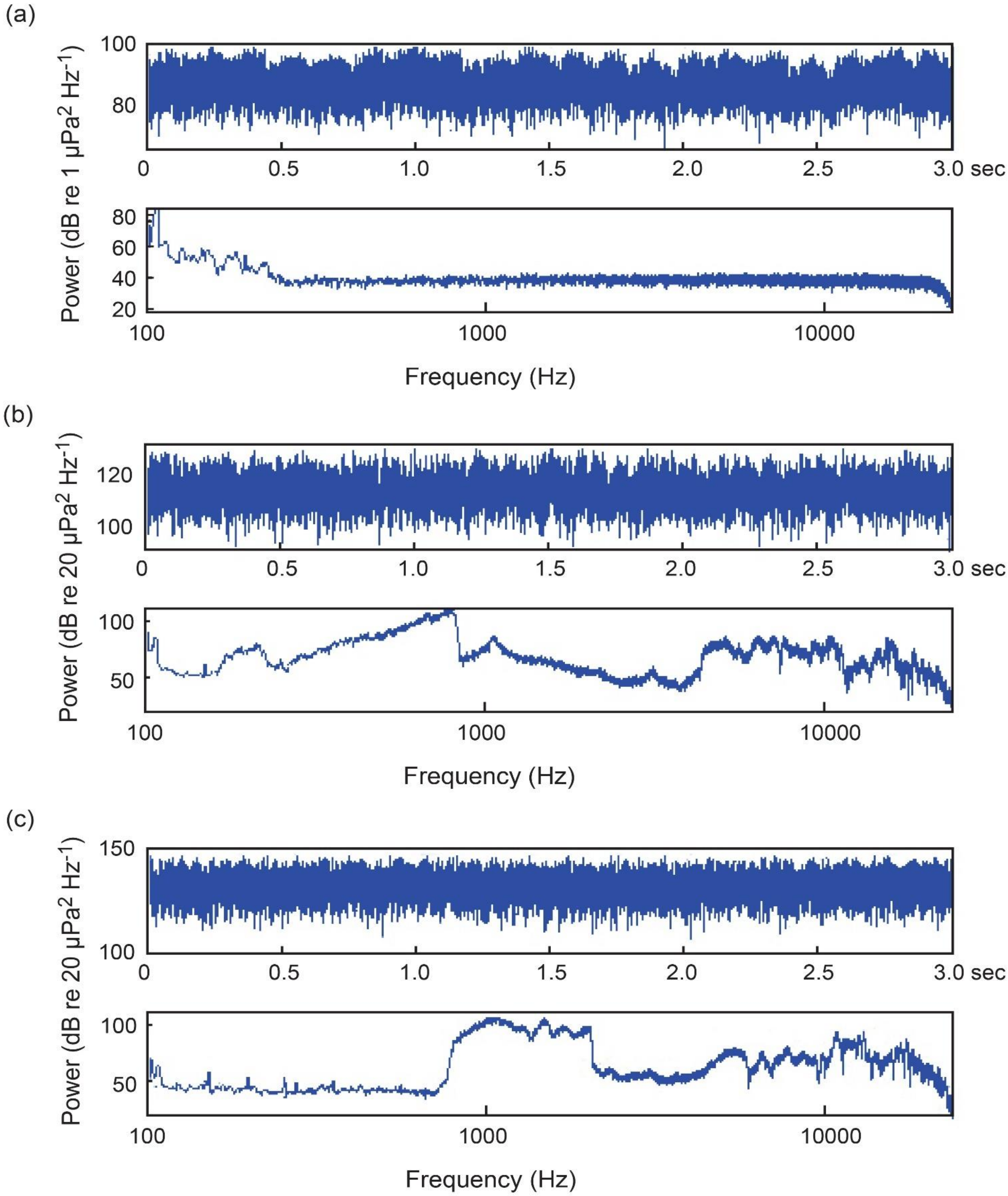

Figure S1. The mean intensity levels measured for baseline (background noise 94.55 dB @ 1 µPa (a); 124.03 dB @ 20 µPa at 124 Hz (b); 140.01 dB @ 20 µPa at 800-2000 Hz (c).
